# Secretory kinase FAM20C triggers adipocyte dysfunction, inciting insulin resistance and inflammation in obesity

**DOI:** 10.1172/JCI191075

**Published:** 2025-10-28

**Authors:** Ankit Gilani, Benjamin D. Stein, Anne Hoffmann, Renan Pereira de Lima, Elizabeth E. Ha, Edwin A. Homan, Lunkun Ma, Alfonso Rubio-Navarro, Tint Tha Ra Wun, Gabriel Jose Ayala Carrascal, Bhavneet Bhinder, Adhideb Ghosh, Falko Noé, Olivier Elemento, Christian Wolfrum, Matthias Blüher, James C. Lo

**Affiliations:** 1 Division of Cardiology, Department of Medicine, Weill Center for Metabolic Health, Cardiovascular Research Institute, and; 2 Department of Medicine, Weill Cornell Medicine, New York, New York, USA.; 3 Helmholtz Institute for Metabolic, Obesity, and Vascular Research, Helmholtz Center Munich, University of Leipzig and University Hospital Leipzig, Leipzig, Germany.; 4 Englander Institute of Precision Medicine, Weill Cornell Medicine, New York, New York, USA.; 5 Institute of Food, Nutrition and Health, ETH Zurich, Switzerland.; 6 Medical Department III — Endocrinology, Nephrology, Rheumatology, University of Leipzig Medical Center, Leipzig, Germany.

**Keywords:** Cell biology, Metabolism, Adipose tissue, Diabetes, Obesity

## Abstract

Obesity is a major driver of type 2 diabetes (T2D) and related metabolic disorders, characterized by chronic inflammation and adipocyte dysfunction. However, the molecular triggers initiating these processes remain poorly understood. We identified FAM20C, a serine/threonine kinase, as an early obesity-induced mediator of adipocyte dysfunction. *Fam20c* expression was substantially upregulated in adipocytes in response to obesity, correlating with a proinflammatory transcriptional signature. Forced expression of *Fam20c* in adipocytes promoted robust upregulation of proinflammatory cytokines and induced insulin resistance that is dependent on its kinase activity. Conversely, deletion of adipocyte *Fam20c* after established obesity and hyperglycemia improved glucose tolerance, augmented insulin sensitivity, and reduced visceral adiposity, without altering body weight. Phosphoproteomic studies revealed that FAM20C regulates phosphorylation of intracellular and secreted proteins, modulating pathways critical to inflammation, metabolism, and ECM remodeling. We identified FAM20C-dependent substrates, such as CNPY4, whose phosphorylation contributes to proinflammatory adipocyte signaling. Of translational relevance, we showed that in humans, visceral adipose *FAM20C* expression positively correlates with insulin resistance. Our findings establish FAM20C as an early regulator of obesity-induced adipocyte dysfunction and systemic metabolic impairment. Our studies provide proof of concept that inhibition of FAM20C may serve as a potential therapy for T2D by restoring adipocyte health.

## Abstract 2

FAM20C is identified as a key mediator of obesity-induced adipocyte dysfunction and inflammation, suggesting its inhibition as a potential therapy for type 2 diabetes.

## Graphical abstract

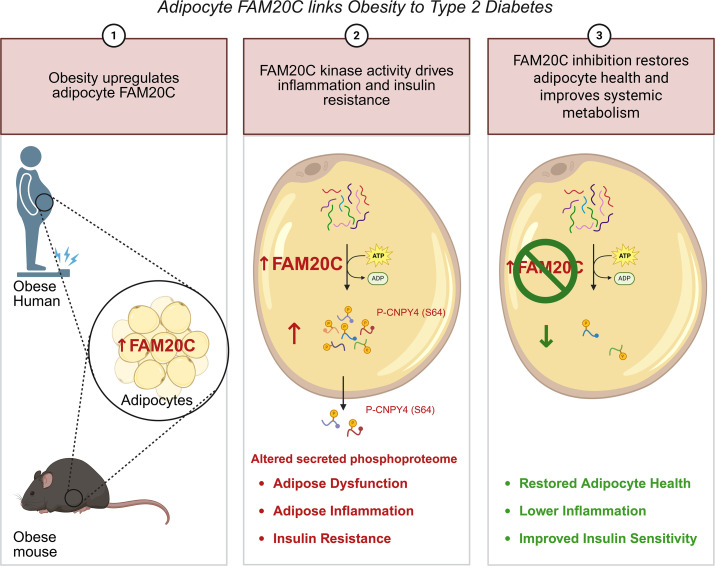

## Introduction

Obesity and type 2 diabetes (T2D) have reached epidemic proportions globally, profoundly impacting public health by increasing the risk for comorbidities including cardiovascular diseases, cancer, kidney and liver dysfunction, and retinal damage (1–4). Central to T2D development is obesity, which induces a cascade of metabolic derangements, notably in adipose tissues (ATs) (5, 6). Adipocytes regulate metabolism by storing excess energy and releasing adipokines that control insulin sensitivity and inflammation (6, 7). However, obesity induces a state of adipocyte dysfunction involving increased production of prodiabetic adipokines such as RBP4, decreased secretion of the antidiabetic adipokines adiponectin and adipsin, insulin resistance, release of toxic metabolites, diminished thermogenic capacity, and chronic inflammation (8–15). Although adipocyte dysfunction is a known driver of obesity-associated metabolic diseases such as T2D, the molecular mediators initiating this process remain poorly understood. Kinases such as c-Jun NH2-terminal kinase (JNK) (16) and noncanonical IκB kinases including IκB kinase ε and TANK-binding kinase 1 (TBK1) (17–19) have been implicated in adipocyte inflammation and insulin sensitivity, highlighting a critical role of protein phosphorylation in development of adipocyte dysfunction.

FAM20C (family with sequence similarity 20, member C), also known as Dentin Matrix Protein 4, is a Golgi-localized and secreted serine/threonine protein kinase (20). It phosphorylates secretory pathway proteins with S-x-E/pS motifs and regulates biomineralization of bones and teeth (21–23). FAM20C contributes to the extracellular phosphoproteome and is implicated in biological functions such as wound healing (24), cell adhesion and migration (25–27), endocytosis (28), and lipoprotein receptor binding (29).

Obesity is often associated with hyperglycemia, hypertension, dyslipidemia, and some cancers, which are key features of metabolically unhealthy obesity (MUO). However, a subset of obese individuals, with what is termed metabolically healthy obesity (MHO), have a substantially lower risk of developing these typical cardiometabolic diseases compared with those with MUO (30–32). This paradox suggests that adipocyte function plays a critical role in determining whether obesity leads to metabolic dysfunction, rather than the mere accumulation of fat. Adipocyte dysfunction is observed in MUO, whereas individuals with MHO maintain better adipocyte function with low inflammation (31, 32), though the underlying mechanisms remain unclear. Identifying molecular mediators initiating the process of adipocyte dysfunction could explain the heterogeneity of cardiometabolic risk factor prevalence in obesity.

Here, we investigated the molecular mechanisms underlying adipocyte dysfunction and identified the kinase FAM20C as a critical regulator of this process. Our data support a model where FAM20C serves as an early mediator of obesity-induced inflammatory signaling and insulin resistance in adipocytes, contributing to the progression from obesity to insulin resistance and T2D. We demonstrate that *Fam20c* expression is upregulated in adipocytes in response to obesity and that its kinase activity drives a proinflammatory gene expression signature. Importantly, KO of *Fam20c* in adipocytes after established obesity and hyperglycemia corrected glucose intolerance and insulin resistance, suggesting that targeting *Fam20c* could be a potential strategy for restoring adipocyte function and metabolic homeostasis. Our phosphoproteomic studies reveal substrates and pathways regulated by adipocyte FAM20C that may contribute to its pathophysiological actions. By identifying *Fam20c* as a key molecular switch in adipocyte dysfunction, our findings provide insights into the mechanisms driving obesity-related metabolic diseases and offer potential therapeutic avenues for preventing or treating T2D.

## Results

### Obesity induces Fam20c, a serine/threonine kinase, in adipocytes.

**Figure 1:**
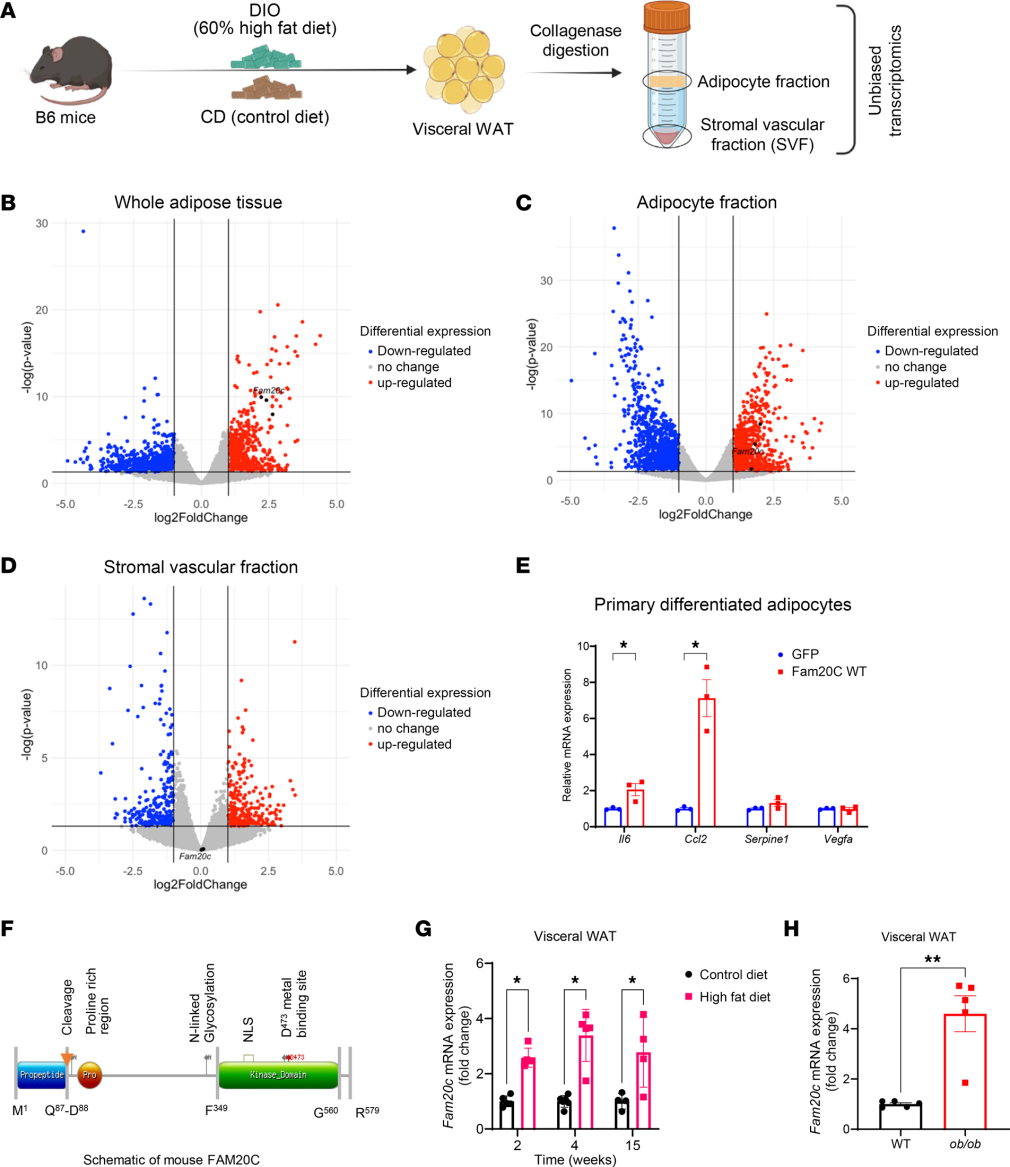
FAM20C is an obesity-induced serine/threonine kinase in adipocytes. (**A**) Schematic of VIS WAT collection from diet-induced obesity (DIO) model of B6/J mice and its fractionation into adipocyte and stromal vascular compartments. (**B**) Volcano plot of DEGs from whole AT, (**C**) adipocyte fraction, and (**D**) SVFs of HFD- versus CD-fed B6/J mice. (**E**) Relative mRNA expression of proinflammatory genes in primary adipocytes of B6/J transduced with respective viral constructs (*n* = 3 per group). (**F**) Schematic of mouse FAM20C protein with highlighted key domains and catalytic sites. NLS, nuclear localization signal. (**G**) Fam20c mRNA expression from VIS WAT of CD- and HFD-fed B6/J mice at respective time points (*n* = 4 per group). (**H**) Fam20c mRNA expression from VIS WAT of 6-week-old WT B6/J and leptin-deficient *ob/ob* mice on CD (*n* = 5 per group). *P < 0.05, **P < 0.01; unpaired, 2-tailed Student’s *t* test. Data are shown as the mean ± SEM.

To identify adipocyte-derived factors that drive early obesity and T2D, we assessed AT, adipocyte, and stromal vascular fraction (SVF) in the well-established B6 model of diet-induced obesity (Figure 1A). B6 WT mice were placed on a 60% high-fat diet (HFD) or regular chow diet (CD) for 4 weeks. Unbiased transcriptomic analyses on visceral (VIS) white adipose tissue (WAT) revealed over 200 genes induced in response to obesity (Figure 1B). Pathway analysis of differentially expressed genes (DEGs) revealed enrichment of cholesterol biosynthesis and metabolism, focal adhesion, and PI3K/AKT/mTOR signaling pathways (Supplemental Figure 1A; supplemental material available online with this article; https://doi.org/10.1172/JCI191075DS1). We also performed an unbiased transcriptomic analysis of the adipocyte fraction and the SVF from these mice (Figure 1, C and D). Similar to the whole AT, pathways associated with focal adhesion and PI3K/AKT/mTOR signaling were also enriched in the adipocyte fraction (Supplemental Figure 1B), whereas pathways for P53 signaling, oxidative stress, and redox pathways were enriched in SVF (Supplemental Figure 1C). To identify adipocyte-specific factors that initiate dysfunction, we performed a preliminary screening of the top candidate genes highly induced in adipocytes in obesity. Lentiviral vector transduction of these factors in primary differentiated adipocytes identified *Fam20c* as a substantial inducer (2- to 6-fold) of *Il6* and *Ccl2* (Figure 1E), indicating a potential role in promoting adipose inflammation. Beyond its transcriptional effects, *Fam20c* was prioritized for further study based on its biochemical classification as a secreted protein and a kinase, both categories of proteins previously implicated in adipose inflammation and dysfunction. We found that *Fam20c* expression was robustly induced early in obesity (Figure 1B) and was specific to the adipocyte fraction (Figure 1C), as *Fam20c* expression was unchanged in the SVF (Figure 1D). These data indicate that *Fam20c* is specifically induced in adipocytes within WAT in response to obesity. Figure 1F shows a schematic of mouse FAM20C, a 579-aa protein, with its kinase domain, a putative propeptide, a proline-rich region, and N-linked glycosylation sites. The kinase domain contains the D473 metal-binding site essential for its enzymatic activity.

*Fam20c* expression was 2.5-, 3.4-, and 2.8-fold higher at 2, 4, and 15 weeks of HFD feeding, respectively, indicating an early and sustained induction throughout obesity (Figure 1G). *Fam20c* was similarly induced in the subcutaneous (SC) WAT and brown adipose tissue (BAT) depots of HFD-fed mice compared with CD-fed mice (Supplemental Figure 1, D and E). We also examined basal expression of *Fam20c* in various tissues from chow-fed WT mice, including BAT, SC WAT, VIS WAT, bone, heart, muscle, liver, and brain. Expression of *Fam20c* in adipose depots was comparable with that in bone, a mineralized tissue previously reported to have high *Fam20c* expression (Supplemental Figure 1F) (33). Moreover, *Fam20c* was upregulated in ATs but not in other metabolic tissues such as liver (Supplemental Figure 1, G–I). It is possible that the increased *Fam20c* in HFD-fed mice results from nutrient composition (e.g., lipid content) of the diet rather than obesity. VIS WAT of genetically obese *ob/ob* mice fed a CD demonstrated 5-fold higher *Fam20c* expression compared with WT controls, suggesting that obesity rather than specific dietary factors drives *Fam20c* induction (Figure 1H). These findings establish *Fam20c* as an early obesity-induced kinase expressed in adipocytes.

The strong induction of *Fam20c* in adipocytes during obesity led us to examine whether this regulation was linked to specific adipocyte subpopulations by analyzing single-cell and single-nucleus RNA-seq datasets of mouse and human WAT (34). In mice, *Fam20c* was upregulated across all 6 adipocyte subclusters following HFD, with the strongest induction in mAd1–3 and mAd6 (Supplemental Figure 2A). In human WAT, *FAM20C* expression was substantially enriched in the hAd5 subcluster, and the proportion of hAd5 cells positively correlated with BMI, suggesting an association between *FAM20C* expression and obesity-driven expansion of specific adipocyte populations (Supplemental Figure 2B). Together, these results suggest that *Fam20c* upregulation is a conserved, obesity-associated feature of specific adipocyte subtypes in both mice and humans.

### Fam20c induces a broad proinflammatory gene expression pattern and insulin resistance in adipocytes.

**Figure 2:**
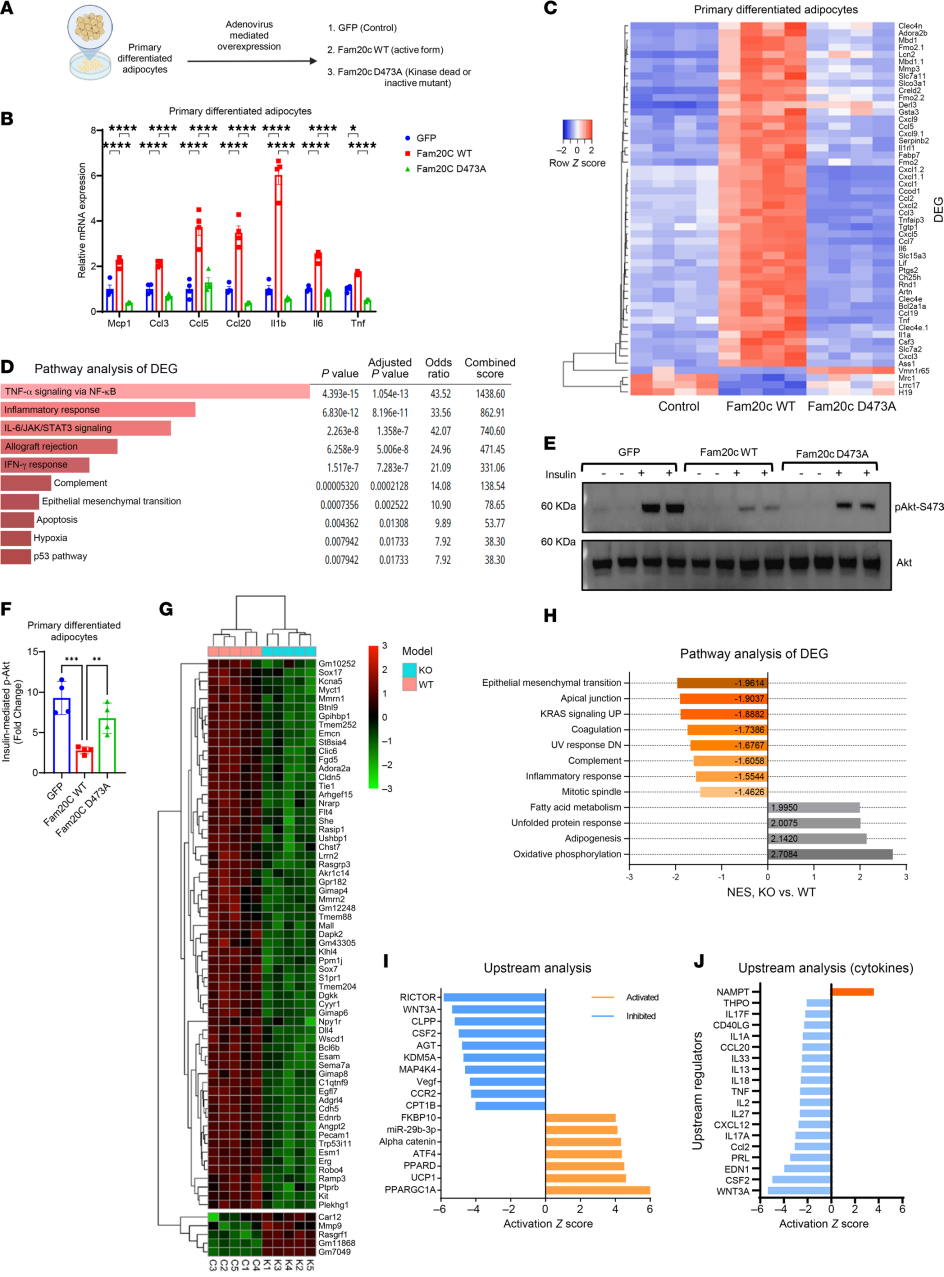
Induction of adipocyte Fam20c elicits a proinflammatory gene expression signature. (**A**) Schematic of viral transduction of primary differentiated adipocytes with respective constructs. (**B**) Relative mRNA expression of proinflammatory genes in primary adipocytes transduced with respective viral constructs (*n* = 4 per group). (**C**) Heatmap of DEGs in primary adipocytes transduced with respective viral constructs (*n* = 4 per group). (**D**) Hallmark pathway analysis of top DEGs between Fam20c WT and Fam20c D473A (kinase-dead mutant) groups. (**E**) Representative Western blot images and (**F**) quantification of insulin-mediated p-AKT S473 induction in primary adipocytes transduced with respective viral constructs and stimulated with either PBS or insulin (10 nM for 10 minutes) (*n* = 4 per group). Three independent experiments were performed. (**G**) Heatmap of DEGs in primary differentiated adipocytes from control and Ad-Fam20c–KO mice (*n* = 4 per group). (**H**) Hallmark pathway analysis of DEGs between primary differentiated adipocytes from control and Ad-Fam20c–KO mice. NES, normalized enrichment score. (**I**) Upstream analysis showing top activated and inactivated pathways and (**J**) upstream cytokine pathways based on DEGs in primary differentiated adipocytes from control and Ad-Fam20c–KO mice. An activation *z* score of ≥2 was used as the cutoff. *P < 0.05, **P < 0.01, ***P < 0.001, ****P < 0.0001; 2-way ANOVA followed by Bonferroni’s multiple-comparison test for **B** and 1-way ANOVA for **F**. Data are shown as the mean ± SEM.

We hypothesized that *Fam20c* in adipocytes may promote adipose inflammation and adipocyte dysfunction to trigger metabolic impairment and T2D. To assess the effect of FAM20C kinase in adipocytes, we overexpressed *Fam20c* in primary differentiated adipocytes using adenovirus carrying the mouse *Fam20c* WT gene (Figure 2A and Supplemental Figure 2, C and D). Forced expression of *Fam20c* resulted in a substantial (2- to 6-fold) increase in chemokine and cytokine genes, including *Ccl2*, *Ccl5*, *Il1b*, and *Tnf* (Figure 2B). To ascertain if these effects require FAM20C’s kinase activity, we engineered a catalytically inactive mutant (D473A) of FAM20C (Figure 2A). By contrast, the *Fam20c* *D473A* mutant did not elicit an increase in chemokine and cytokine expression (Figure 2B). To determine the full extent of the effects of *Fam20c* on adipocytes, we performed unbiased transcriptomic analyses on primary adipocytes transduced with *Fam20c*. The majority of genes regulated in response to *Fam20c* were related to inflammation, indicating that FAM20C preferentially induces a broad proinflammatory expression pattern (Figure 2C). Importantly, the kinase activity of FAM20C was necessary for these effects as the kinase-dead FAM20C mutant failed to elicit the same response as the WT (Figure 2C). Pathway analysis revealed activation of TNF-α signaling, inflammatory response, and IL-6/JAK/STAT3 pathways (Figure 2D). Forced expression of *Fam20c* in the L1 adipocyte cell line recapitulated the inflammatory gene expression seen in primary adipocytes (Supplemental Figure 2E), supporting a direct cell-autonomous role of FAM20C in promoting robust inflammatory gene expression pattern in adipocytes.

Building on the link between *Fam20c* upregulation and adipocyte dysfunction, we investigated whether *Fam20c* directly contributes to insulin resistance. To this end, we performed in vitro insulin sensitivity assays in primary adipocytes overexpressing either WT or catalytically inactive *Fam20c*. Overexpression of *Fam20c* in adipocytes caused a striking approximately 70% reduction in insulin-stimulated AKT phosphorylation compared with GFP control, indicating profound impairment in insulin signaling (Figure 2, E and F). In contrast, the D473A mutant had no effect, indicating that this impairment is dependent on FAM20C’s kinase activity. Together, these findings support a direct, kinase-dependent role for FAM20C in promoting adipocyte inflammation and insulin resistance.

### Identification of Fam20c-dependent transcriptional changes in adipocytes under physiological conditions.

To investigate the role of *Fam20c* in adipocytes, we ablated *Fam20c* specifically in adipocytes by crossing *Fam20c-*floxed and *Adiponectin-Cre* transgenic mice. Primary differentiated adipocytes from control (*Fam20c*-floxed) and Ad-*Fam20c*–KO (*Fam20c-*floxed*/Adiponectin-Cre*) mice were subjected to RNA-seq to identify *Fam20c*-dependent transcriptional alterations. Analysis of DEGs revealed a significant downregulation of 63 transcripts and upregulation of 5 transcripts in the KO adipocytes (*P* < 0.05; Figure 2G). Pathway enrichment analysis revealed suppression of pathways linked to adipocyte dysfunction, including epithelial-mesenchymal transition, apical junction assembly, KRAS signaling, and inflammatory response, whereas pathways associated with enhanced adipocyte function, including fatty acid metabolism, adipogenesis, and oxidative phosphorylation, were upregulated in the adipocytes devoid of *Fam20c* (Figure 2H). Adipocyte dysfunction is often associated with an imbalance of adipokine secretion leading to inflammation and insulin resistance. To determine whether *Fam20c* regulates the adipocyte secretome, we filtered for genes encoding secreted proteins or extracellular space components. Among these, *Esm1* (endothelial cell–specific molecule 1) exhibited the most significant downregulation in KO adipocytes (Supplemental Figure 2F). *Esm1* regulates fatty acid synthesis, cell migration, integrin binding, and angiogenesis (35, 36), suggesting that its decreased expression in KO adipocytes may correspond to reduced immune cell infiltration and inflammation. Expression of several proinflammatory cytokines (*Ccl6*, *Ccl8*, *Cxcl16*, and *Il16*) was also reduced in the KO adipocytes (Supplemental Figure 2G).

Upstream analysis revealed activation of transcription factors associated with improved adipocyte metabolism in KO adipocytes. PPARGC1A was notably activated, a key regulator of AT metabolism and thermogenesis (37) (Figure 2I). Conversely, transcription factors linked to cell growth and inflammatory signaling, such as RICTOR and WNT3A, were inactivated. Upstream cytokine analysis showed substantial inactivation of proinflammatory cytokines, such as TNF, CCL2, and CCL20, in KO adipocytes (Figure 2J). Remarkably, NAMPT, a cytokine associated with improved insulin sensitivity in AT (38), was upregulated in the KO group. These findings suggest that under physiological conditions, adipocyte *Fam20c* drives the activation of proinflammatory cytokines and chemokines, while repressing transcription factors and cytokines linked to enhanced adipocyte function and insulin sensitivity.

### Adipocyte-specific KO of Fam20c in obese and diabetic mice improves glucose metabolism and insulin sensitivity.

To investigate the pathophysiological role of adipocyte *Fam20c*, we generated constitutive (Ad-Fam20c–KO) and inducible (iAd-Fam20c–KO) adipocyte-specific *Fam20c*-KO mice by crossing *Fam20c*-floxed with *Adiponectin-Cre* and *Adiponectin-CreERT* transgenic lines, respectively. When fed a CD, neither model exhibited significant metabolic alterations. In Ad-Fam20c–KO mice, body weight and glucose tolerance were comparable with controls (Supplemental Figure 3, A and B). Similarly, in iAd-Fam20c–KO mice, in which *Fam20c* deletion was induced in 12-week-old adults, body weight and glucose tolerance remained unchanged relative to controls (Supplemental Figure 3, C and D). Furthermore, the weights of major metabolic tissues, including BAT, SC and VIS WAT depots, and liver, were not different between groups (Supplemental Figure 3E).

**Figure 3:**
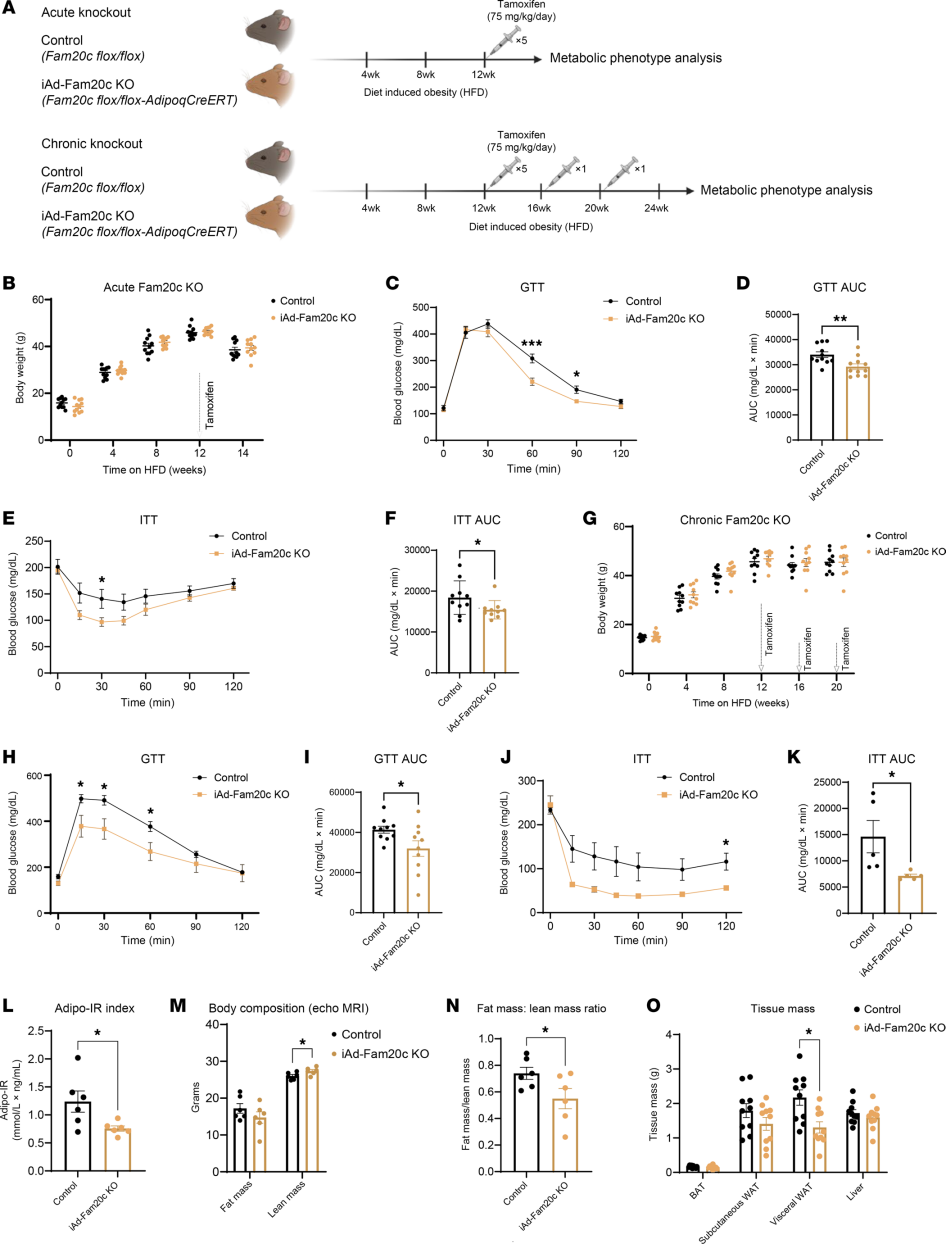
Adipocyte-specific deletion of Fam20c corrects metabolic impairments of diet-induced obesity. (**A**) Schematic of mouse models employing acute (2 weeks) and chronic (12 weeks) KO of adipocyte Fam20c as a disease-modifying therapy for obesity-induced T2D. (**B**) Body weights at different time points, (**C**) glucose tolerance test (GTT), and (**E**) insulin tolerance test (ITT) for control and iAd-Fam20c–KO mice following acute deletion of adipocyte Fam20c (*n* = 11 per group). (**D** and **F**) AUC calculations for **C** and **E**, respectively. (**G**) Body weights at different time points, (**H**) GTT, and (**J**) ITT for control and iAd-Fam20c–KO mice following chronic deletion of adipocyte Fam20c (*n* = 10 per group). (**I** and **K**) AUC calculations for **H** and **J**, respectively. (**L**) Adipo-IR as a marker of adipose insulin sensitivity, (**M**) body composition analysis by EchoMRI, and (**N**) fat mass/lean mass ratio from HFD-fed control and iAd-Fam20c–KO mice following chronic Fam20c deletion (*n* = 6 per group). (**O**) Mass of AT depots and liver from HFD-fed control and iAd-Fam20c–KO mice following chronic Fam20c deletion (*n* = 10 per group). *P < 0.05, **P < 0.01, ***P < 0.001; unpaired, 2-tailed Student’s *t* test for **D**, **F**, **I**, and **K**–**O** and repeated-measure 2-way ANOVA for **C**, **E**, **H**, and **J**. Data are shown as the mean ± SEM.

To determine the potential of FAM20C inhibition as a disease modifying therapy for T2D, control and iAd-*Fam20c*–KO mice were fed HFD for 3 months to induce obesity and insulin resistance. Subsequently, tamoxifen was administered to selectively ablate adipocyte *Fam20c* (Figure 3A and Supplemental Figure 3, F and G). Following acute *Fam20c* deletion (2 weeks after injection), body weights were not significantly different between the groups (39.4 ± 3.5g vs. 38.6 ± 3.5g for controls) (Figure 3B). Acute deletion of *Fam20c* significantly improved glucose tolerance in iAd-*Fam20c*–KO mice compared with controls (Figure 3, C and D). Additionally, insulin tolerance tests revealed that iAd-Fam20c–KO mice displayed enhanced insulin sensitivity relative to controls (Figure 3, E and F). These findings suggest that acute deletion of adipocyte *Fam20c* ameliorates glucose homeostasis and insulin resistance in the context of diet-induced obesity.

To further evaluate sustained effects of adipocyte *Fam20c* deletion on glucose metabolism, we extended the study to determine longer-term effects of FAM20C disruption. Control and iAd-*Fam20c*–KO mice were maintained on HFD for an additional 12 weeks with monthly tamoxifen injections to maintain *Fam20c* KO (Figure 3A). Although body weight was not changed (Figure 3G), chronic deletion of adipocyte *Fam20c* continued to confer substantial metabolic benefits. Both glucose tolerance and insulin sensitivity continued to improve by approximately 25% and >100%, respectively, compared with controls (Figure 3, H–K). These results indicate that the therapeutic benefits of adipocyte *Fam20c* deletion on glucose metabolism and insulin sensitivity are sustained over an extended period of metabolic stress. Additionally, we assessed AT insulin sensitivity using the adipose tissue insulin resistance index (adipo-IR) index, which integrates plasma free fatty acids (FFAs) and insulin. The adipo-IR index was approximately 40% lower in iAd-*Fam20c*–KO mice compared with controls (0.75 ± 0.04 vs. 1.24 ± 0.46, *P* = 0.03), suggesting that *Fam20c* deletion ameliorates AT insulin resistance (Figure 3L). Taken together, these data support the hypothesis that adipocyte *Fam20c* deletion enhances glucose metabolism and insulin sensitivity, providing a potential therapeutic avenue for improving metabolic function in established T2D.

### Adipocyte-specific KO of Fam20c results in a reduction of VIS WAT mass.

**Figure 4:**
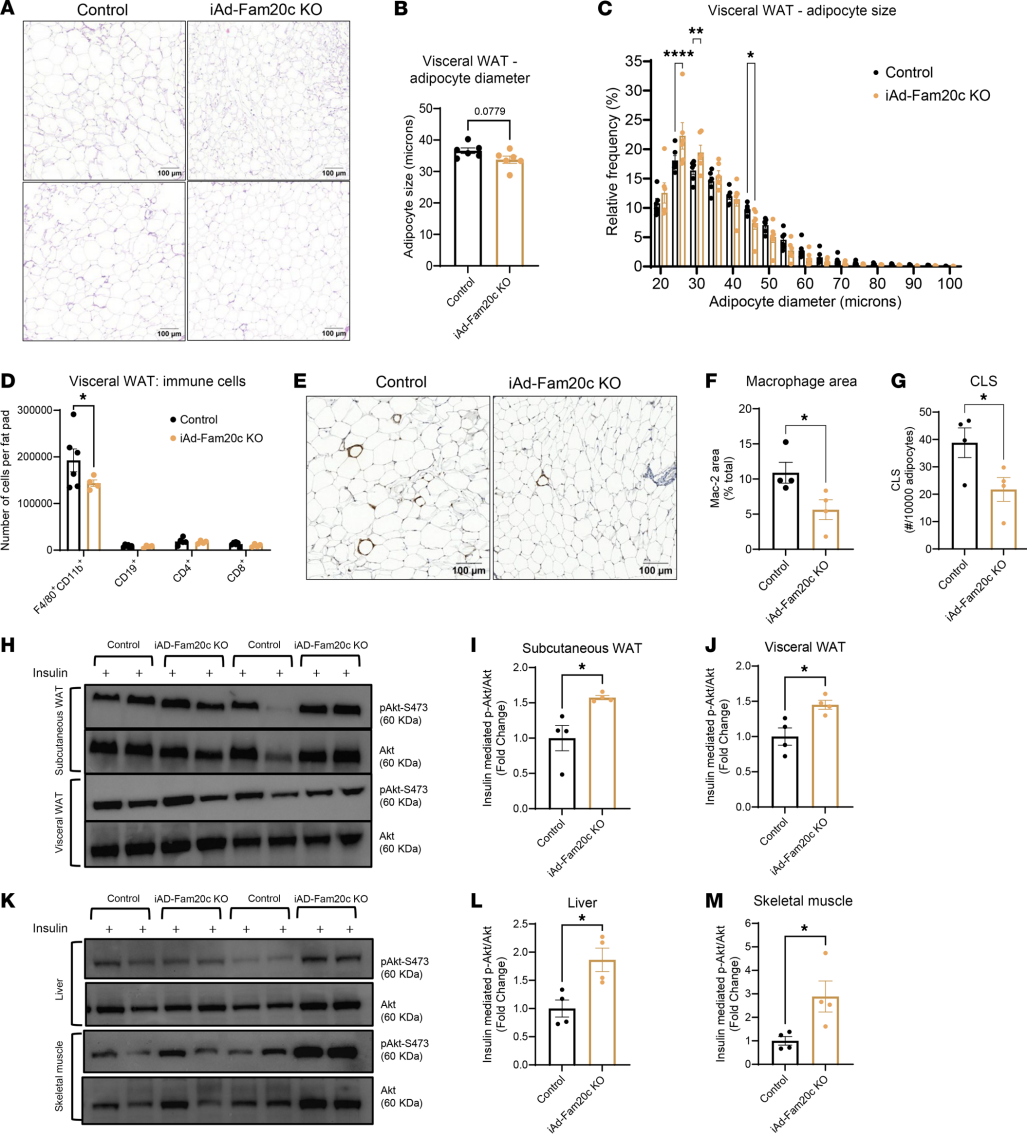
Adipocyte-specific deletion of Fam20c shifts adipocyte size distribution toward smaller adipocytes, decreases ATMs, and improves insulin sensitivity. (**A**) Representative images of H&E-stained VIS WAT sections of HFD-fed control and iAd-Fam20c–KO mice following chronic Fam20c deletion. Scale bars: 100 μm. (**B**) Mean adipocyte diameter and (**C**) frequency size distribution for adipocyte size in HFD-fed control and iAd-Fam20c–KO mice following chronic Fam20c deletion (*n* = 6 per group). (**D**) Number of ATMs (F4/80^+^CD11b^+^), B cells (CD19^+^), CD4^+^ cells, and CD8^+^ cells in control and iAd-Fam20c–KO mice fed HFD for 4 weeks followed by acute Fam20c deletion (*n* = 4–6 per group). (**E**) Representative images of Mac-2 staining, (**F**) quantification of macrophage (Mac-2–stained) area represented as percentage of total adipose area, and (**G**) quantification of CLSs represented as CLS per 10,000 adipocytes from VIS WAT sections of control and iAd-Fam20c–KO mice fed HFD for 4 weeks followed by acute Fam20c deletion (*n* = 4 per group). Scale bars: 100 μm. (**H**) Representative Western blot images for insulin-mediated p-AKT stimulation in SC and VIS WAT of control and iAd-Fam20c–KO mice fed HFD for 4 weeks followed by acute Fam20c deletion. (**I** and **J**) Quantifications of p-AKT/AKT from **H** for SC and VIS WAT, respectively (*n* = 4 per group). (**K**) Representative Western blot images for insulin-mediated p-AKT stimulation in liver and skeletal muscle of control and iAd-Fam20c–KO mice fed HFD for 4 weeks followed by acute Fam20c deletion. (**L** and **M**) Quantifications for p-AKT/AKT from **K** for liver and skeletal muscle, respectively. *P < 0.05, **P < 0.01, ****P < 0.0001; unpaired, 2-tailed Student’s *t* test. Data are shown as the mean ± SEM.

To assess the impact of adipocyte *Fam20c* deletion on body composition, we conducted metabolic and histological analyses in control and iAd-*Fam20c*–KO mice with chronic *Fam20c* deletion. EchoMRI analysis revealed that iAd-*Fam20c*–KO mice exhibited a mild but significant increase in lean mass (27.3 ± 0.4 g vs. 26 ± 0.4 g, *P* = 0.04) compared with controls, while total fat mass was unchanged (14.7 ± 1.5 g vs. 17.2 ± 1.3 g, *P* = 0.25), suggesting a redistribution of body mass (Figure 3M). Consistent with this, the fat/lean mass ratio was significantly lower in iAd-*Fam20c*–KO mice (0.55 ± 0.07 vs. 0.74 ± 0.11, *P* = 0.02 for controls), indicating a decrease in overall adiposity relative to lean tissue (Figure 3N). Additionally, iAd-*Fam20c*–KO mice displayed a striking 40% selective reduction in VIS WAT mass compared with controls, with no significant differences observed in BAT or SC WAT (Figure 3O). To investigate whether the observed reduction in VIS WAT mass was associated with changes in adipocyte morphology, we performed histological analysis of H&E-stained sections (Figure 4A). While the average adipocyte diameter was unchanged (Figure 4B), frequency distribution analysis revealed a significant shift in adipocyte size distribution. Specifically, iAd-*Fam20c*–KO mice exhibited a significantly higher proportion of smaller adipocytes and a correspondingly lower proportion of larger adipocytes (Figure 4C). This shift in adipocyte size distribution likely contributes to the reduced VIS WAT mass. To assess for adipose inflammation, we stained for macrophage markers and quantified crown-like structures (CLSs) in the VIS WAT. Numbers of adipose tissue macrophages (ATMs) and CLSs were not different between control and iAd-*Fam20c*–KO mice (Supplemental Figure 4, A–C), indicating that chronic *Fam20c* deletion following HFD feeding may not be sufficient to reverse CLS formation and associated proinflammatory changes.

Collectively, these findings demonstrate that *Fam20c* regulates VIS WAT expansion and adipocyte size distribution, and its deletion protects against VIS adiposity and obesity-induced insulin resistance.

### Adipocyte Fam20c promotes early inflammatory remodeling and insulin resistance during diet-induced obesity.

To define the contribution of adipocyte *Fam20c* to immune cell infiltration during early obesity, we disrupted adipocyte FAM20C after 4 weeks of HFD using inducible iAd-Fam20c–KO and control mice. Masses of AT depots and livers remained unchanged between control and iAd-Fam20c–KO mice (Supplemental Figure 4D). However, flow cytometry analysis of the VIS WAT SVF revealed a 25% reduction in F4/80^+^CD11b^+^ ATMs in iAd-Fam20c–KO mice compared with controls (Figure 4D and Supplemental Figure 4E). VIS WAT tissue B cells as well as CD4^+^ and CD8^+^ cells were unchanged between the groups (Figure 4D). Consistent with this, immunohistochemistry of VIS WAT revealed that iAd-Fam20c–KO mice had 49% lower macrophage area and 44% lower CLS compared with controls (Figure 4, E–G). These results indicate that adipocyte FAM20C facilitates early macrophage infiltration and proinflammatory remodeling of VIS WAT during obesity onset.

We next examined whether *Fam20c* influences insulin signaling in peripheral tissues during early obesity. Control and iAd-Fam20c–KO mice received HFD for 4 weeks followed by acute *Fam20c* deletion and then were challenged in vivo with insulin. Insulin-stimulated AKT phosphorylation (p-AKT Ser473) was measured in SC and VIS WAT, liver, and skeletal muscle (Figure 4, H–M). Early and acute adipocyte ablation of *Fam20c* in the course of obesity resulted in substantial improvements in insulin signaling with elevations in insulin-stimulated p-AKT by 1.6-fold in SC WAT, 1.5-fold in VIS WAT, 1.9-fold in liver, and 2.9-fold in skeletal muscle compared with controls, indicating enhanced insulin responsiveness in multiple metabolic tissues (Figure 4, H–M). Together, these findings suggest that *Fam20c* facilitates early adipose inflammation and systemic insulin resistance during the initial stages of diet-induced obesity.

### FAM20C regulates phosphorylation of intracellular and secreted proteins in adipocytes, modulating inflammatory and metabolic pathways.

**Figure 5:**
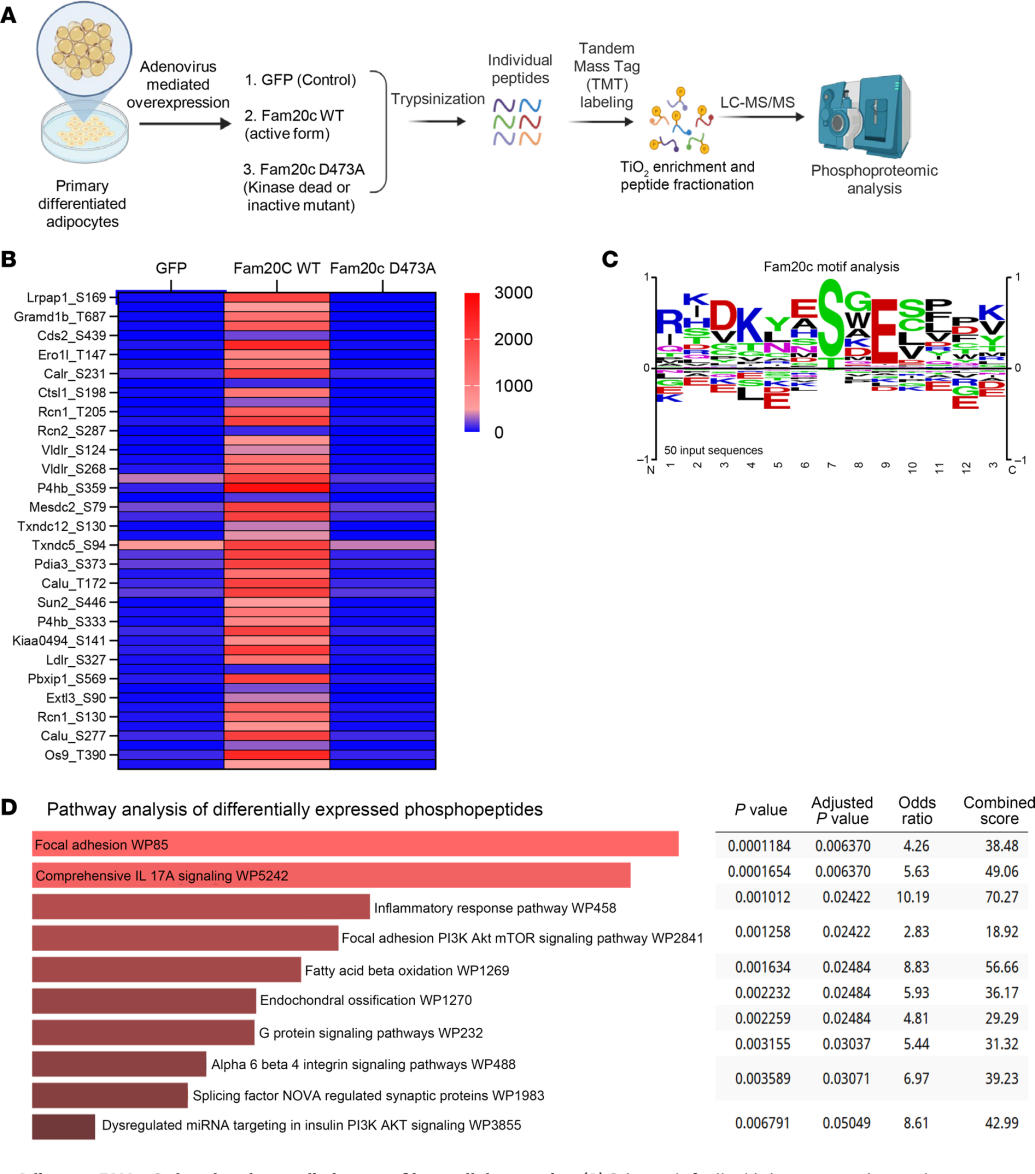
Adipocyte FAM20C phosphorylates a distinct set of intracellular proteins. (**A**) Schematic for liquid chromatography–tandem mass spectrometry–based (LC-MS/MS–based) unbiased phosphoproteomic analysis of intracellular proteins from primary differentiated adipocytes transduced with respective viral constructs. (**B**) Heatmap showing top differentially phosphorylated proteins in primary adipocytes transduced with respective viral constructs. (**C**) Motif analysis and sitemap of top identified FAM20C-dependent phosphosites in **B**. (**D**) Pathway analysis of differentially expressed phosphopeptides from primary adipocytes transduced with either a WT construct of Fam20c or a kinase-dead mutant (D473A).

FAM20C is a serine/threonine kinase localized to the Golgi apparatus that phosphorylates secreted proteins (21). Since the kinase-dead D473A mutant of FAM20C did not elicit an inflammatory gene expression phenotype, we hypothesized that FAM20C actions in adipocytes are mediated via protein phosphorylation. We performed unbiased phosphoproteomics on primary adipocytes transduced with either *Fam20c* WT, the kinase-dead mutant (*D473A*), or a control (*Gfp*) virus (Figure 5A). Forced expression of *Fam20c* WT led to the identification of a distinct set of >500 phosphorylated peptides, which were absent in cells overexpressing the *Fam20c D473A* mutant, confirming the kinase-dependent nature of these phosphopeptides (Figure 5B). Motif analysis revealed that a majority of these phosphosites exhibited a S-x-E consensus sequence, a known motif specifically targeted by FAM20C among secretory pathway kinases (Figure 5C). Pathway enrichment analysis of FAM20C-regulated phosphopeptides identified involvement of focal adhesion, PI3K/AKT/mTOR signaling, and inflammatory response pathways (Figure 5D), implicating that FAM20C regulates key processes involved in cellular metabolism, immune response, and protein trafficking in adipocytes.

**Figure 6:**
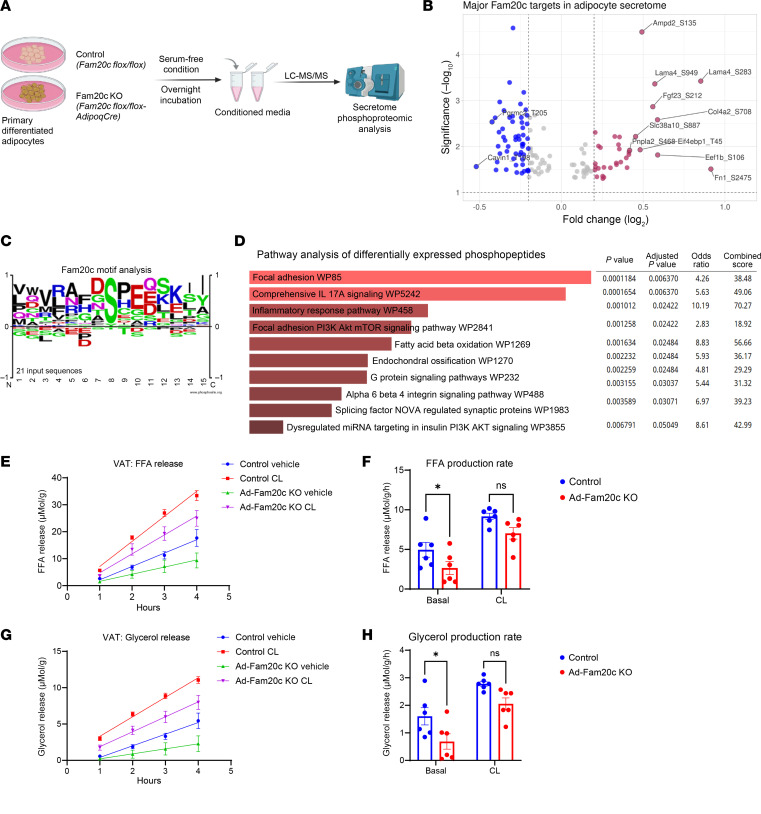
Adipocyte FAM20C phosphorylates a distinct set of secreted proteins. (**A**) Schematic of LC-MS/MS–based unbiased phosphoproteomic analysis of secreted proteins from control and Fam20c-deficient primary adipocytes. (**B**) Volcano plot showing top differentially phosphorylated secreted proteins in primary adipocytes with and without Fam20c. (**C**) Motif analysis and sitemap of top identified FAM20C-dependent phosphosites in **B**. (**D**) Pathway analysis of differentially expressed secreted phosphoproteins in primary adipocytes with and without Fam20c. (**E**) FFA release plotted over time and (**F**) rate of FFA production per hour from VAT explants of HFD-fed control and Ad-Fam20c–KO mice treated with either vehicle or 0.5 μM CL-316243 (*n* = 6 per group). (**G**) Glycerol release plotted over time and (**H**) rate of glycerol production per hour from VAT explants of HFD-fed control and Ad-Fam20c–KO mice treated with either vehicle or 0.5 μM CL-316243 (*n* = 6 per group). *P < 0.05; 2-way ANOVA followed by Šidák’s post hoc multiple-comparison test. Data are shown as the mean ± SEM.

Next, to explore the role of FAM20C in modulating phosphorylation of adipocyte secretome, we conducted phosphoproteomics on conditioned media of primary differentiated adipocytes derived from control and Ad-*Fam20c*–KO mice (Figure 6A). Secreted phosphopeptides enriched by Fam20c included LAMA4-Ser283/Ser949, FGF23-Ser212, FN1-Ser2475, and COL4A2-Ser708, all ECM proteins implicated in fibrosis (Figure 6B). Most sites conformed to the FAM20C consensus motif of S-x-E (Figure 6C). Pathway analysis of these secreted phosphopeptides also revealed enrichment of focal adhesion, PI3K/AKT/mTOR, and integrin signaling (Figure 6D). These findings suggest that FAM20C-mediated phosphorylation regulates the secretion of ECM proteins and signaling molecules that contribute to adipocyte dysfunction, potentially through mechanisms linked to fibrosis and inflammation. Additionally, we observed that FAM20C phosphorylated Patatin-like phospholipase domain–containing protein 2 (PNPLA2, also known as ATGL) at Ser468 (Figure 6B). PNPLA2 plays a pivotal role in the initiation of triglyceride hydrolysis, suggesting that FAM20C may regulate adipocyte lipolysis through PNPLA2. To investigate the impact of FAM20C on adipose lipolysis, we conducted an ex vivo lipolysis assay using VIS WAT explants from either control or Ad-Fam20c–KO mice after 8 weeks of HFD. Basal release of FFA and glycerol was reduced by approximately 45% and 50%, respectively, in KO explants compared with controls, consistent with improved metabolic regulation following Fam20c deletion (Figure 6, E–H). Stimulation with the β3-adrenoceptor agonist CL-316,243 (0.5 μM) induced similar lipolytic responses in both groups (Figure 6, E–H). These findings are consistent with the established observation that obesity is associated with elevated basal lipolysis due to the blunted antilipolytic effect of insulin and catecholamine resistance (39–41). Our results suggest that ablation of adipocyte FAM20C reduces basal lipolysis in obesity, likely due to improved insulin sensitivity, without affecting catecholamine responsiveness.

Together, these data provide strong evidence that FAM20C is a critical kinase in adipocytes, influencing both intracellular and secreted protein phosphorylation. By regulating key pathways involved in focal adhesion, PI3K/AKT/mTOR signaling, inflammatory response, and lipolysis, FAM20C modulates adipocyte function and contributes to insulin resistance in obesity.

### Obesity-induced Fam20c in the VIS WAT phosphorylates proteins involved in adipogenesis and AT dysfunction.

**Figure 7:**
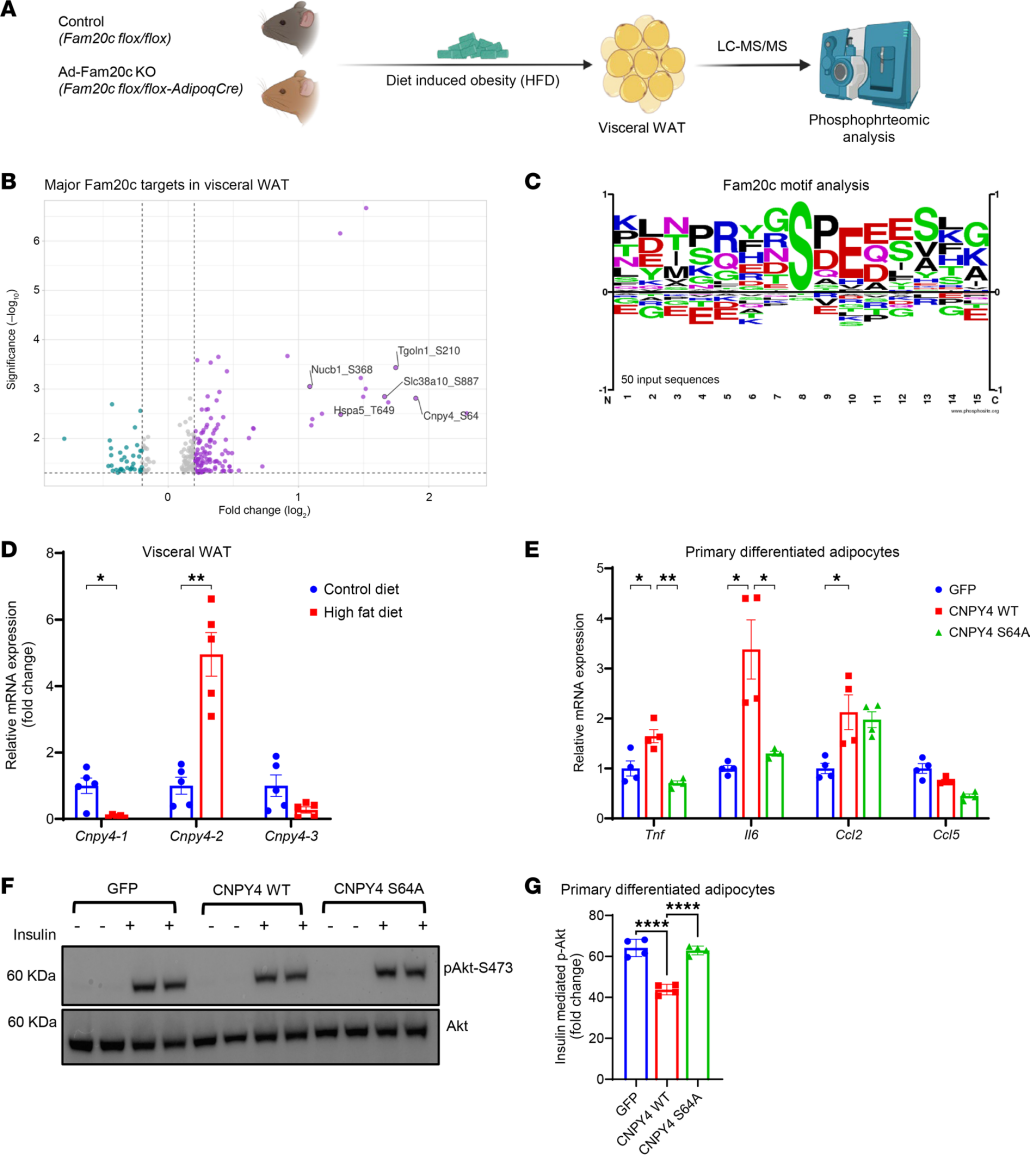
Obesity-induced FAM20C in VIS WAT phosphorylates proteins, causing AT dysfunction. (**A**) Schematic of LC-MS/MS–based unbiased phosphoproteomic analysis of VIS WAT from HFD-fed control and Ad-Fam20c–KO mice. (**B**) Volcano plot showing top differentially phosphorylated proteins in VIS WAT of control versus Ad-Fam20c–KO mice. (**C**) Motif analysis and sitemap of top identified FAM20C-dependent phosphosites in **B**. (**D**) Relative expression of mouse Cnpy4 transcript variant 1 (*Cnpy4-1*), transcript variant 2 (*Cnpy4-2*), and transcript variant 3 (*Cnpy4-3*) in B6 WT mice fed either CD or HFD for 12 weeks (*n* = 5 per group). (**E**) Relative mRNA expression of proinflammatory genes in primary adipocytes transduced with respective viral constructs (*n* = 4 per group). (**F**) Representative Western blot images and (**G**) quantification of insulin-mediated p-AKT S473 induction in primary adipocytes transduced with respective viral constructs and stimulated with either PBS or insulin (10 nM for 10 minutes) (*n* = 4 per group). Three independent experiments were conducted. *P < 0.05, **P < 0.01, ****P < 0.0001; 2-tailed Student’s *t* test for **D**, 2-way ANOVA for **E**, and 1-way ANOVA for **G**. Data are shown as the mean ± SEM.

To identify pathological substrates of FAM20C in vivo, we performed phosphoproteomics on VIS WAT of control and Ad-*Fam20c*–KO mice on a HFD for 8 weeks to increase FAM20C expression in controls (Figure 7A). This experimental setup generated a high signal/noise ratio, allowing comprehensive assessment of FAM20C’s role in AT dysfunction during obesity. A volcano plot revealed significant changes in phosphorylation patterns between the 2 groups (Figure 7B), with most phosphosites conforming to the S-x-E motif, confirming FAM20C specificity (Figure 7C). Pathway analysis of differentially phosphorylated proteins identified enrichment in integrin-mediated cell adhesion, insulin signaling, and regulation of the actin cytoskeleton, suggesting a critical role for FAM20C in these processes under pathological conditions of obesity (Supplemental Figure 5A).

A key substrate of FAM20C identified through phosphoproteomics was Canopy FGF signaling regulator 4 (CNPY4), phosphorylated at Ser64 in a FAM20C-dependent manner (Figure 7B). CNPY4 is a secreted protein shown to regulate the cell surface expression of TLR4, which propagates the production of proinflammatory cytokines (42, 43). This prompted us to investigate CNPY4’s role in mediating the effect of FAM20C on AT inflammation. Notably, mouse *Cnpy4* encodes 3 isoforms, and variant-specific qPCR revealed a distinct expression pattern in VIS WAT during obesity. Isoform 2 was markedly upregulated, isoform 1 was downregulated, and isoform 3 remained unchanged at low expression levels (Figure 7D). We focused subsequent mechanistic studies on isoform 2 due to its obesity-inducible expression. To test the hypothesis that CNPY4 phosphorylation by FAM20C promotes inflammation, we overexpressed CNPY4 WT and CNPY4 S64A mutant in primary differentiated adipocytes (Figure 7E and Supplemental Figure 5, B and C). Adipocytes overexpressing CNPY4 WT exhibited a significant increase in expression of proinflammatory cytokines, including *Tnf*, *Il6*, and *Ccl2*, compared with controls (Figure 7E). In contrast, adipocytes overexpressing the S64A mutant, which lacks the FAM20C phosphorylation site, did not show any increase in inflammatory gene expression of *Tnf* and *Il6*. *Ccl2* is a FAM20C-regulated cytokine but was not regulated by its phosphorylation of CNPY4, indicating that CNPY4 may induce *Ccl2* by a FAM20C-independent mechanism. Another FAM20C-regulated cytokine *Ccl5* was also unaffected, suggesting CNPY4 mediates part of FAM20C’s actions on adipose inflammation.

To investigate whether CNPY4 also mediates insulin resistance downstream of FAM20C, we performed in vitro insulin signaling assays. Following insulin stimulation, primary adipocytes overexpressing WT CNPY4 exhibited significantly reduced AKT phosphorylation (p-AKT Ser473) compared with controls, consistent with impaired insulin signaling (Figure 7, F and G). In contrast, overexpression of the CNPY4 S64A mutant did not impair p-AKT induction by insulin, indicating that FAM20C-dependent phosphorylation of CNPY4 at Ser64 is required to impair insulin signaling. Taken together, these results suggest that FAM20C-mediated phosphorylation of CNPY4 at Ser64 promotes proinflammatory gene expression and contributes to AT insulin resistance in obesity. These findings also highlight the importance of FAM20C in regulating key signaling proteins involved in adipocyte inflammation and dysfunction in obesity.

### Adipose FAM20C levels in humans positively correlate to insulin resistance.

**Figure 8:**
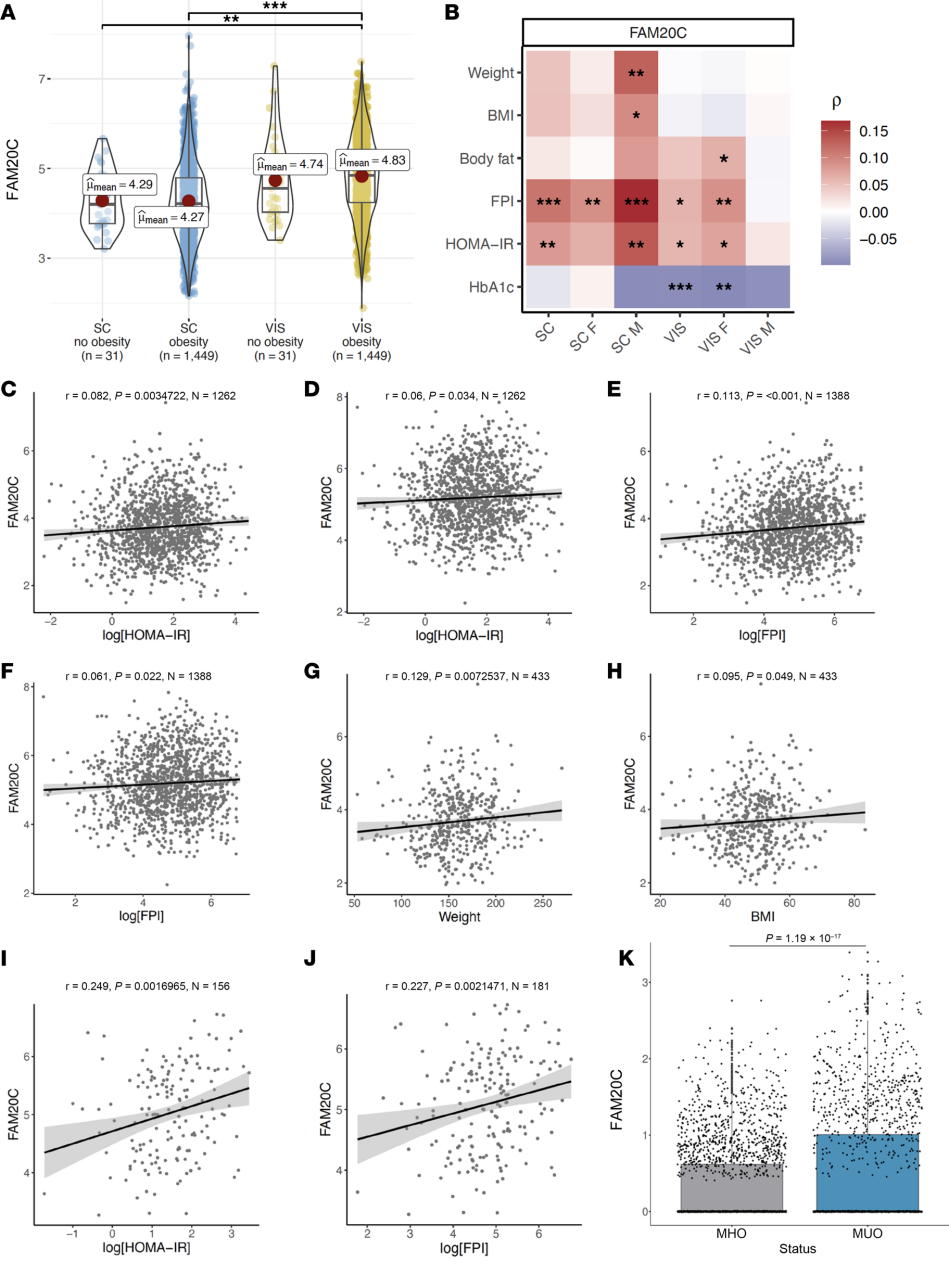
Adipose FAM20C expression in humans positively correlates with insulin resistance. (**A**) FAM20C gene expression comparison for SC and VIS ATs for patient subgroups with and without obesity. The box plots depict the minimum and maximum values (whiskers), the upper and lower quartiles, and the median. (**B**) FAM20C gene correlation analysis with metabolic parameters. (**C**) Correlation of SC FAM20C gene expression with HOMA-IR. (**D**) Correlation of VIS FAM20C gene expression with HOMA-IR. (**E**) Correlation of SC FAM20C gene expression with FPI. (**F**) Correlation of VIS FAM20C gene expression with FPI. (**G**) Correlation of SC FAM20C gene expression with body weight for males. (**H**) Correlation of VIS FAM20C gene expression with BMI for males. (**I**) Correlation of VIS FAM20C gene expression with HOMA-IR in individuals not receiving antihyperglycemic medications. (**J**) Correlation of VIS FAM20C gene expression with FPI in individuals not receiving antihyperglycemic medications. (**K**) VIS adipocyte FAM20C expression from single-nucleus RNA-seq study in MHO and MUO individuals. *P < 0.05, **P < 0.01, ***P < 0.001; Welch’s 1-way ANOVA with Games-Howell post hoc test for **A**, Spearman’s correlation coefficient analysis with a confidence interval of 0.95 for **B**–**J**, and 2-tailed Student’s *t* test for **K**.

To investigate the relationship between *FAM20C* expression and metabolic dysfunction in humans, we analyzed *FAM20C* expression in a cross-sectional cohort comprising paired omental (VIS) and abdominal (SC) ATs from 1,480 individuals in the Leipzig Obesity Biobank (LOBB). *FAM20C* expression was significantly higher in VIS than in SC ATs in individuals with obesity (Figure 8A). However, no significant differences in *FAM20C* were observed between individuals with and without obesity in either VIS or SC AT, though this could be underpowered due to the small number of individuals without obesity (*N* = 31). We then examined the correlation between *FAM20C* expression and various metabolic parameters, including body weight, BMI, body fat percentage, fasting plasma insulin (FPI), homeostasis model assessment of insulin resistance (HOMA-IR), and HbA1c (Figure 8B). In both VIS and SC ATs, *FAM20C* levels positively correlated with HOMA-IR, a well-established indicator of insulin resistance (Figure 8, C and D). Additionally, *FAM20C* expression showed significant positive correlation with FPI, suggesting a state of hyperinsulinemia in association with insulin resistance (Figure 8, E and F). These findings suggest a potential role for adipose FAM20C in the development of insulin resistance in humans.

In males, *FAM20C* levels in SC AT also correlated positively with body weight and BMI (Figure 8, G and H), suggesting that higher *FAM20C* expression in this depot may be linked to adiposity. To control for the potential confounding effects of antidiabetic medications, we excluded patients receiving glucose lowering treatments, including insulin, metformin, DPP-4 inhibitors, sulfonylureas, and glitazones (Supplemental Figure 6, A and B). In this subset, we observed a strong positive correlation between VIS AT *FAM20C* levels and both FPI and HOMA-IR (Figure 8, I and J). However, no significant correlations were found for *FAM20C* levels in SC AT (Supplemental Figure 6, C and D), further supporting the notion that VIS *FAM20C* may be a key contributor to the development of insulin resistance in obesity. Taken together, these data suggest that elevated *FAM20C* in VIS AT is strongly associated with insulin resistance and hyperinsulinemia in individuals with obesity. In contrast, the correlation between *FAM20C* and metabolic parameters appears to be weaker in SC AT, highlighting the specific role of VIS *FAM20C* in metabolic dysfunction. VIS adipose remodeling is a hallmark of MUO and is closely linked to AT dysfunction and systemic insulin resistance. To investigate *FAM20C* expression in this pathological phenotype, we reanalyzed the publicly available single-nucleus RNA-seq dataset profiling VAT from individuals with either MHO or MUO (44). Our analysis revealed 60% higher *FAM20C* expression in VIS adipocytes from MUO individuals compared with MHO counterparts (*P* = 1.19 × 10^−^¹^7^) (Figure 8K and Supplemental Figure 6E). These results identify elevated FAM20C expression as a molecular feature of MUO-associated adipocytes, reinforcing its role in the development of metabolic dysfunction in obesity.

## Discussion

The global epidemic of obesity and T2D is fueled by a complex pathogenesis involving inflammatory and metabolic disturbances within AT, particularly adipocyte dysfunction. Despite extensive research on the processes associated with adipocyte dysfunction, critical gaps remain in understanding its molecular triggers. Our study identifies *Fam20c*, an obesity-induced gene, as an early mediator of adipocyte dysfunction, unveiling its ability to alter both intracellular and extracellular signaling within AT. Ablation of adipocyte FAM20C also enhanced insulin sensitivity in other metabolic tissues, such as the liver and muscle, placing FAM20C within ATs as a systemic mediator of insulin resistance. By linking FAM20C kinase to adipocyte inflammation, adipose insulin resistance, and systemic metabolic impairment, we expand the current understanding of molecular pathways that transition obesity into T2D, highlighting adipocyte FAM20C as a potential therapeutic target.

A key finding of our study is that *Fam20c* expression is significantly and selectively induced in adipocytes in 2 mouse models of obesity and T2D with different dietary compositions. This induction is unique to adipocytes, as the SVF did not exhibit a similar upregulation. Prior research has demonstrated cell-type–specific transcriptional responses to metabolic stress in obesity (34, 45). Notably, a single-cell study on mouse WAT from Emont et al. revealed *Fam20c* as a marker of an adipocyte subcluster (mAd4) that increased with HFD (34). This study also revealed an obesity-associated increase in *Fam20c* across all 6 mouse adipocyte subclusters (mAd1–mAd6) and 1 human adipocyte subcluster (hAd5) that positively correlated with BMI. Sustained induction of *Fam20c* across multiple stages of obesity underscores its potential role as an early and persistent driver of adipocyte dysfunction. Dietary components of HFD have been shown to influence the degree of metabolic impairment in mouse models (46, 47). The elevation of *Fam20c* in genetically obese *ob/ob* mice, independent of HFD, emphasizes its role as a response to obesity rather than to dietary lipid exposure, though it is possible that additional nutritional components may induce *Fam20c*.

Our study further demonstrates that FAM20C’s kinase activity is crucial for promoting inflammation and adipocyte dysfunction. Overexpression of WT *Fam20c* in primary adipocytes induced a robust proinflammatory gene expression profile and triggered insulin resistance, whereas its kinase-dead mutant (D473A) did not. While other kinases, such as JNK (16, 48) and TBK1 (17–19), have been implicated in adipose inflammation and dysfunction, these are typically activated by proinflammatory cytokine signaling. Notably, TBK1 can suppress inflammation by attenuating NF-κB (17) and depends on prior adipose inflammation for its activation in obesity (19). In contrast, FAM20C acts as an early mediator of adipocyte dysfunction by directly inducing proinflammatory cytokine signaling, making it a potential target for addressing the initial causes of adipocyte dysfunction.

Pathways linked to TNF-α and IL-6/JAK/STAT3 signaling are well-established contributors to adipose inflammation and systemic insulin resistance (49, 50). Our transcriptomic data from primary adipocytes indicate that *Fam20c* induction upregulates these pathways. Conversely, *Fam20c* KO leads to downregulation of these pathways and a concomitant upregulation of pathways involved in fatty acid metabolism, adipogenesis, and oxidative phosphorylation. Oxidative phosphorylation is often impaired in obese adipocytes, with diminished mitochondrial activity leading to inflammation, insulin resistance, and adipocyte dysfunction (51, 52). Interestingly, the oxidative phosphorylation pathway was highly activated in *Fam20c-*KO adipocytes along with upstream activation of PPARGC1A, a key transcription factor driving mitochondrial biogenesis and thermogenesis. This further supports the notion that *Fam20c* promotes a state of metabolic dysfunction by inhibiting pathways enhancing adipocyte metabolism.

A recent report showed that mice with constitutive KO of adipocyte *Fam20c* are protected from diet-induced obesity and have slightly improved glucose homeostasis under HFD (53). With the mild protection from obesity, subtle improvements in glucose tolerance were expected in the constitutive adipocyte *Fam20c* KO. It was unclear if deletion of adipocyte *Fam20c* can reverse established obesity and T2D. That previous report also lacked molecular insight into how FAM20C promotes diabetes and obesity (53). In our study, we used a tamoxifen-inducible model to ablate adipocyte *Fam20c* after a HFD feeding regimen with similar body weights before and after tamoxifen. We did not observe differences in body weights with postdevelopment deletion of *Fam20c*, which differs from the other study (53). The basis for the protection from diet-induced obesity remains unknown and could be due to postnatal development issues with adipocyte *Fam20c* ablation. Importantly, we found that time-restricted targeting of adipocyte *Fam20c* in established T2D remodeled the adipose tissue and enhanced insulin sensitivity. We found no significant changes in the number of ATMs or CLSs with chronic deletion of adipocyte *Fam20c*. There may be changes in other immune cell compartments or macrophage subsets with later loss of Fam20C. However, deletion of adipocyte *Fam20c* following short-term HFD decreases ATM populations and CLSs, indicating *Fam20c* promotes inflammatory adipose remodeling during early obesity. Importantly, the improvements in glucose homeostasis strengthened over 3 months, suggesting that deletion of *Fam20c* in adipose tissue may be effective as a disease modifying therapy for T2D. These findings highlight that adipocyte-specific *Fam20c* deletion confers sustained metabolic benefits. We also found that adipocyte-specific deletion of *Fam20c* improved insulin sensitivity across other metabolic organs, including liver and skeletal muscle. The mechanism(s) and the quantitative improvements in insulin sensitivity across tissues with loss of adipocyte FAM20C are still unknown and will be the subject of future research. Overall, our data not only validate *Fam20c* as a key mediator of insulin resistance in adipocytes but also suggest that inhibition of *Fam20c* may serve as a promising therapeutic approach to alleviate obesity-induced metabolic dysfunction.

One striking finding from this study was a marked reduction in VIS WAT mass in iAd-*Fam20c*–KO mice, coupled with a shift in adipocyte size distribution toward smaller adipocytes. VIS WAT is a critical depot in obesity-associated metabolic diseases, as it is particularly susceptible to inflammatory expansion and dysfunction (54). Our results suggest that *Fam20c* plays a pivotal role in regulating VIS WAT expansion in response to obesity. The reduction in VIS WAT mass, despite no changes in SC tissue or BAT, suggests a potential depot-specific role of *Fam20c* in mediating adipose dysfunction. Moreover, this effect was not attributable to the degree of deletion, as all 3 depots displayed significant KO. Further mechanistic studies are needed to elucidate the pathways by which *Fam20c* selectively impairs obesity-associated VIS WAT expansion. The shift toward smaller adipocytes in the absence of *Fam20c* likely reflects reduced adipocyte hypertrophy, which may limit the severity of VIS WAT dysfunction typically observed in obesity. Obesity is frequently linked to increased basal lipolysis and attenuated catecholamine-stimulated lipolysis (39–41). In our study, VIS WAT from Ad-Fam20c–KO mice exhibited lower basal lipolysis, with no alteration in stimulated lipolysis. This phenotype may be attributed to either enhanced insulin sensitivity in the AT or a direct modulation of the adipocyte triglyceride lipase PNPLA2 activity. Our phosphoproteomic analysis identified FAM20C-dependent phosphorylation of PNPLA2 at Ser468, suggesting a potential regulatory role of FAM20C in lipolysis. Further studies are required to elucidate the functional implications of this phosphorylation event and its broader effects on lipid metabolism.

Our phosphoproteomic analysis identified several putative FAM20C targets, both intracellular and secreted, highlighting its multifaceted role in adipocyte dysfunction. We observed FAM20C-dependent phosphorylation of CNPY4 at Ser64, which appears critical for amplifying inflammatory responses within adipocytes. Overexpression of WT CNPY4 enhanced proinflammatory cytokine production and impaired insulin sensitivity, whereas its phosphorylation-deficient mutant (S64A) did not. This provides a direct mechanistic link between FAM20C kinase activity and adipocyte dysfunction, positioning CNPY4 as a downstream effector of FAM20C. Furthermore, we report an obesity-associated, isoform-specific change in *Cnpy4* expression in VIS WAT, with isoform 2 being selectively induced, suggesting that posttranscriptional regulation of *Cnpy4* may further shape inflammatory and metabolic outputs in obesity. Future in vivo studies are needed to define the role of CNPY4 in insulin resistance and adipose inflammation. While our findings elucidate 1 pathway of FAM20C-mediated dysfunction, they raise important questions about the breadth of its substrate specificity and the interplay between intracellular and secreted phosphoproteins. Intracellularly, FAM20C-dependent phosphorylation of other substrates, such as SLC38A10, could regulate adipocyte amino acid transport to modulate inflammation and insulin sensitivity. Extracellularly, FAM20C-dependent phosphorylation of ECM components such as FN1 and LAMA4 suggests a broader role in tissue remodeling, fibrosis, and chronic inflammation, hallmarks of VIS WAT dysfunction​. Further investigation into these pathways could reveal additional mechanisms through which FAM20C exacerbates metabolic disease.

Previous studies in liver (HepG2), breast cancer (MDA-MB-231), and osteosarcoma (U-2 OS) cell lines demonstrated that FAM20C is a critical regulator of the phosphosecretome (21). However, in our adipocyte secretome data, only a few secreted proteins were phosphorylated by FAM20C, indicating a cell-type–specific effect. Prior work also showed that FAM20C phosphorylates plasma proteins, although these studies were conducted in vitro using conditioned media from cancer cell line models (21). Additional phosphoproteomic analyses of plasma are needed to identify FAM20C substrates in circulation. Furthermore, studies employing the ER-TurboID tag in Ad-*Fam20c*–KO mice are essential to assess how adipocyte FAM20C contributes to the phosphorylation of plasma proteins. These investigations will help elucidate the mechanisms through which FAM20C might alter the plasma phosphoproteome, potentially exerting autocrine or paracrine effects that influence whole-body insulin sensitivity.

Our study also provides important clinical insights into the relationship between high adipose *FAM20C* expression and insulin resistance. There is pronounced heterogeneity in cardiometabolic risk among individuals with obesity, and those with MUO are known to exhibit altered gene expression in metabolic tissues including adipose and skeletal muscle (30–32, 44). Petersen et al. reported decreased expression of genes involved in inflammation and ECM remodeling in SC AT from MUO individuals (31), but they did not examine VIS AT. Utilizing a single-nucleus RNA-seq study of VIS AT, we found that VIS adipocyte *FAM20C* is markedly elevated in MUO (44). In our study, *FAM20C* expression was markedly higher in VIS AT compared with in SC AT, and its levels positively correlated with insulin resistance markers such as HOMA-IR and FPI. These correlations were particularly strong in individuals not receiving glucose-lowering medications, suggesting that *FAM20C* is not merely a consequence of metabolic dysregulation but a contributing factor. The preferential association of VIS AT *FAM20C* with metabolic impairment aligns with the notion that VIS AT is more metabolically active and inflammation prone than SC AT, which has a protective role in energy storage​. These data validate the translational relevance of our findings and highlight *FAM20C* as a potential biomarker for identifying individuals with obesity at high risk for T2D. Further studies measuring adipose and plasma levels of FAM20C in larger longitudinal cohorts of control and obese individuals are needed to validate these observations. Overall, our findings pave the way for the development of targeted therapies that modulate FAM20C activity in adipocytes to treat obesity-related metabolic diseases. Future research is crucial to further elucidate the precise molecular targets of FAM20C in adipocytes and to translate these findings into effective therapies for metabolic diseases.

## Methods

### Experimental model and study participant details

#### Sex as a biological variable.

Only male mice were used in the study since we observed spontaneous Cre activation and body weight changes in female *Fam20c-*floxed*/Adiponectin-CreEMy RT* mice in the absence of tamoxifen. We therefore excluded data from female mice to avoid confounding results.

#### In vivo animal studies.

*Fam20*c-floxed mice were a gift from Chunlin Qin (Texas A&M University, Dallas, Texas, USA) (23) and were backcrossed at least 5 generations to C57BL/6J mice. *Adiponectin-Cre* (strain 028020), *Adiponectin-CreERT* (strain 024671), and *ob/ob* (strain 000632) mice were purchased from The Jackson Laboratory. Constitutive and conditional (tamoxifen-inducible) adipocyte-specific *Fam20c*-KO mice were generated by breeding *Fam20c*-floxed homozygous mice with *Adiponectin-Cre* and *Adiponectin-CreERT* mice, respectively. *Fam20c*-floxed mice were used as controls from the same backcross generation. All mice were maintained in plastic cages under a 12-hour-light/12-hour-dark cycle at 22°C with free access to water and food (control diet: PicoLab Rodent Diet 5053, 20% protein, 4.5% fat). For the diet-induced obesity model, 4-week-old male and female mice were placed on a 60% HFD (D12492i, Research Diets) for respective time periods. Tamoxifen was dissolved in corn oil and injected intraperitoneally at 75 mg/kg/day. Fat and lean mass were determined via EchoMRI analysis.

#### Studies involving human participants.

Human data were sourced from LOBB (https://www.helmholtz-munich.de/en/hi-mag/cohort/leipzig-obesity-bio-bank-lobb). This biobank includes paired samples of abdominal SC and omental VIS AT, body fluids, and anthropometric data. Adipose samples were collected during elective laparoscopic surgeries following established protocols (55). Body composition and metabolic parameters were assessed using standardized methods (32, 56). Exclusion criteria included age < 18 years, chronic substance or alcohol misuse, smoking within 12 months, acute inflammatory diseases, glitazone use, end-stage malignancy, >3% weight loss within 3 months, uncontrolled thyroid disorders, and Cushing’s disease. The cross-sectional cohort comprised 1,480 individuals, divided into nonobese (*N* = 31; 52% female; age: 55.8 ± 13.4 years; BMI: 25.7 ± 2.7 kg/m²) and obese (*N* = 1,449; 72% female; age: 46.4 ± 11.7 years; BMI: 49.2 ± 8.3 kg/m²) groups. A smaller subset of this cohort was analyzed excluding those receiving blood glucose–lowering medications (insulin, metformin, DPP-4 inhibitors, glitazones, or sulfonylureas). This subcohort included 203 individuals categorized into nonobese (N = 6; 34% female; age: 47.4 ± 5.7 years; BMI: 25.5 ± 2.9 kg/m²) and obese (N = 197; 68% female; age: 45.8 ± 12.0 years; BMI: 48.5 ± 7.6 kg/m²).

#### Cell lines and primary cultures.

For primary differentiated adipocytes, SVFs from inguinal adipose of 6- to 8-week-old male mice were prepared and differentiated for 6–8 days (57). Primary white adipocytes were cultured in DMEM/F12K media (Gibco, Thermo Fisher Scientific) with 10% FBS at 37°C, 5% CO_2_, until confluent. Differentiation involved 48-hour treatment with 0.5 mM 3-isobutyl-1-methylxanthine, 1 mM dexamethasone, 850 nM insulin, and 1 mM rosiglitazone, followed by 48 hours with 850 nM insulin and 1 mM rosiglitazone and then a further 48 hours with 850 nM insulin. For differentiated 3T3-L1 adipocytes, NIH/3T3 fibroblasts (ATCC, CRL-1658) were grown to 80% confluency and differentiated using the same protocol. To produce lentivirus, the HEK293T cell line (ATCC, CRL-3216) was used.

### Method details

#### Blood chemistry and serum insulin analysis.

Mice were fasted overnight (14–16 hours) for glucose tolerance tests and injected intraperitoneally with d-glucose solution (2 g/kg). For insulin tolerance tests, mice were fasted for 6 hours and injected with 0.5 mIU/kg insulin. Blood glucose was measured with a commercial glucometer (OneTouch) using tail vein blood samples. Plasma insulin levels were measured after 6 hours of fasting. Blood was collected into lithium heparin tubes and centrifuged at 2,000*g* at 4°C, and plasma insulin levels were determined by ELISA (Mercodia). Plasma FFAs were quantified using the NEFA-HR(2) assay kit (Fujifilm).

#### Histological analysis.

AT was immediately perfused with PBS and fixed with 10% neutral-buffered formalin (VWR), then transferred to 70% ethanol. Paraffin-embedding, sectioning, and H&E staining were done by the Memorial Sloan Kettering Cancer Center Laboratory of Comparative Pathology core facility. Slides were imaged using a Zeiss Axioscan7 at ×20 magnification. Adipocyte diameter was measured from H&E-stained VIS WAT sections using ImageJ (NIH) with the Adiposoft plug-in (58). For immunohistochemistry analysis of CLSs, tissue sections were incubated with anti-MAC-2 (BioLegend, 125402) followed by biotinylated and HRP-conjugated rat secondary antibody (Thermo Fisher Scientific, 31830). Histochemical reactions were performed using the Vectastain ABC HRP kit (Vector Labs, PK-4000) and DAB peroxidase substrate kit (Vector Labs, SK-4100). Sections were counterstained with hematoxylin. CLS frequency was calculated as total number of CLSs per 10,000 adipocytes.

#### Viral constructs and transduction.

Adenoviral (pAd/CMV/V5-DEST, V49320, Invitrogen), retroviral (pMSCVpuro, 634401, Clontech), and lentiviral (pCDH-CMV-MCS-EF1-puro, CD510B-1, System Biosciences) expression vectors were used. Mouse *Fam20c* and *Cnpy4* CDS genes were cloned into these constructs. To generate *Fam20c* D473A and *Cnpy4* S64A mutants, the QuikChange II site-directed mutagenesis kit (200555, Agilent) was used. For viral production, packaging cells (Phoenix for retrovirus and 293T for lentivirus) were transfected at 70% confluence by lipofectamine 2000 (Invitrogen) method with 10 μg of respective vectors. After 48 hours, the viral supernatant was harvested and filtered. Cells were incubated overnight with the viral supernatant, supplemented with 8 μg/mL polybrene. Subsequently, puromycin (Thermo Fisher Scientific) was used for selection.

#### RNA extraction and real-time qPCR.

Total RNA from AT and adipocytes was isolated using the RNeasy Mini Kit (Qiagen). A total of 1 μg RNA was reverse transcribed using a high-capacity cDNA RT kit (Thermo Fisher Scientific). qPCR was performed using SYBR Green Master Mix (Quanta) and gene-specific primers on the QuantStudio 6 Flex system (Thermo Fisher Scientific). Relative mRNA levels were determined by normalizing to ribosomal protein S18 using the ΔΔC_T_ method. Primer sequences are listed in the Supplemental Table 1.

### In vitro insulin sensitivity assay

Primary differentiated adipocytes were washed with PBS and incubated overnight in serum-free low-glucose DMEM with 0.5% BSA. Cells were stimulated with either 0 or 10 nM insulin for 10 minutes. Media was removed and cells lysed with RIPA buffer for protein extraction and Western blot analysis.

### In vivo insulin sensitivity assay

Mice were fasted for 4 hours, injected with either PBS or 5 U/kg insulin intraperitoneally, and sacrificed 10 minutes after injection. ATs, liver, and skeletal muscle were harvested for assessment of p-AKT and AKT protein by Western blotting.

### Flow cytometry

Epididymal (VIS) AT was digested for 20 minutes in buffer containing collagenase D, Dispase-II, DNase-I, and calcium chloride. Cell suspensions were filtered through 100 μm then 40 μm filters, and the SVF was collected by centrifugation at 500*g* for 10 minutes. Cells were incubated with CD16/32 (Fc block, BioLegend, 101302) for 15 minutes. Cells were then stained with anti-CD45.2 (BioLegend, 109824) for hematopoietic cells, anti-F4/80 (BioLegend, 123110) for macrophages, anti-CD11b (BioLegend, 101206) for macrophages, anti-CD19 (BioLegend, 115520) for B cells, and anti-CD4 (BD Biosciences, 553650) and anti-CD8 (BD Biosciences, 553032) for T cells. DAPI stain (BioLegend, 422801) was used to mark dead cells. Cells were analyzed using a Sony MA900 cell sorter and FlowJo software.

### Western blot analysis

Cells or tissues were lysed in RIPA buffer with protease and phosphatase inhibitors. Protein extracts were resolved on NuPAGE Bis-Tris gels (Thermo Fisher Scientific) and transferred to PVDF membranes. Membranes were incubated overnight (4°C) with primary antibodies including FAM20C (Proteintech, 25395-I-AP), p-AKT Ser473 (Cell Signaling, 9271S), AKT (Cell Signaling, 9272S), CNPY4 (AF5015, R&D Systems), and Actin-HRP (Life Technologies, MA515739HRP). Detection of proteins was carried out by incubations with HRP-conjugated secondary antibodies followed by enhanced chemiluminescence detection reagents. Band intensity was quantified using Fiji/ImageJ (NIH).

#### Unbiased transcriptomics.

Unbiased transcriptomic analyses of whole VIS WAT, adipocyte fraction, SVFs, and primary differentiated adipocytes transduced with adenovirus were performed using the Affymetrix GeneChip Mouse Genome 430A 2.0 Array (Thermo Fisher Scientific) according to the manufacturer’s protocol.

#### RNA-seq and pathway analysis.

After RNA isolation, RNA integrity was analyzed using an Agilent 2100 bioanalyzer, and concentrations were measured by NanoDrop spectrophotometer (Thermo Fisher Scientific). Preparation of the RNA library was performed by the Genomics Core at Weill Cornell Medicine using the SMARTer v4 Ultra Low Input RNA Kit (Clontech, 63488) and Nextera XT DNA Library Preparation Kit (Illumina). The normalized cDNA libraries were pooled and sequenced on an Illumina HiSeq 4000 sequencer at 50 pair-end cycles. Sequencing reads were mapped with STAR v2.6.0c to the mouse reference genome (GRCm38.p6) (59). Fragments per gene were counted with featureCounts v1.6.2 with respect to Ensembl annotation 33137190. DEGs between pairwise comparisons were identified by Wald’s tests using DESeq2 v1.26.080, with Benjamini-Hochberg–corrected 2-tailed *P* values < 0.05 considered statistically significant (60). Biological analyses, including canonical pathways, biological processes, or transcription factors, were performed using Ingenuity Pathway Analysis (Qiagen). Log_2_-transformed counts per million values were used for heatmap plots of bulk RNA-seq data, which were centered and scaled by row.

### Proteomic sample preparation and LC-MS3 analysis

Intracellular and secretome phosphoproteomic sample preparation was conducted as previously described (61). The detailed methodology for phosphoproteomic experiments can be found in Supplemental Methods.

#### Human adipose RNA-seq.

RNA was extracted from AT using the SMART-seq protocol (62). Single-end sequencing of all libraries was performed on a NovaSeq 6000 at the Functional Genomics Center Zurich. The raw sequencing reads underwent adapter and quality trimming with Fastp v0.20.0 (63), applying a minimum read length of 18 nucleotides and a quality threshold of 20. Read alignment to the human reference genome (assembly GRCh38.p13, GENCODE release 32) and gene-level expression quantification were executed using Kallisto v0.48 (64). Samples with read counts > 20 million were downsampled to this threshold using ezRun v3.14.1 (https://github.com/uzh/ezRun, commit ID 860b8d7; accessed on March 23, 2022). Data normalization was performed using a weighted trimmed mean of the log expression ratios, with adjustments made for age, sex, and transcript integrity numbers. All analyses were carried out in R v4.3.1 (www.R-project.org).

### Statistics

All statistical analyses were performed using GraphPad Prism 9. Unpaired 2-tailed *t* tests, unpaired Welch’s *t* test, and 2-way ANOVA were used. *P* < 0.05 was considered statistically significant. All data were assessed for normality and found to be normally distributed to support the application of parametric statistical tests. Data in bar graphs are shown as mean ± SEM. Details on individual statistical tests and number of samples (*N*) are indicated in respective figure legends.

### Study approval

All animal studies were approved by the Institutional Animal Care and Use Committee and Research Animal Resource Center at Weill Cornell Medicine. The human study received ethical approval from the Ethics Committee of the University of Leipzig (approval no. 159-12-21052012) and was conducted in accordance with the principles outlined in the Declaration of Helsinki. All participants provided written informed consent before being included in the study.

### Data availability

The main data supporting the findings of this study are available within the article and its supplemental files, including the Supporting Data Values file. The human RNA-seq data from LOBB have not been deposited in a public repository due to restrictions imposed by patient consent but can be obtained from MB upon request. Mouse RNA-seq data are available in the Gene Expression Omnibus under accession number GSE309948. All derived MS/MS data and metadata are publicly available on MassIVE (accession MSV000099484) and ProteomeXchange (accession PXD069449).

## Author contributions

Conceptualization: A Gilani and JCL. Methodology: A Gilani, JCL, BDS, AH, BB, OE, EAH, LM, ARN, TTRW, RPDL, EEH, and GJAC. Investigation: A Gilani, EAH, LM, ARN, TTRW, RPDL, and EEH. Visualization: A Gilani, JCL, BDS, and AH. Funding acquisition: A Gilani, JCL, EAH, EEH, and MB. Project administration: A Gilani and JCL. Supervision: A Gilani and JCL. Writing (original draft): A Gilani and JCL. Writing (review and editing): A Gilani, BDS, EAH, LM, ARN, TTRW, RPDL, BB, OE, AH, A Ghosh, FN, CW, and MB.

## Funding support

This work is the result of NIH funding, in whole or in part, and is subject to the NIH Public Access Policy. Through acceptance of this federal funding, the NIH has been given a right to make the work publicly available in PubMed Central.

- NIH grants R01 DK121140 and R01 DK121844 (JCL).
- American Diabetes Association postdoctoral fellowship 9-22-PDFPM-01 (AG).
- Tri-I StARR National Institute of Allergy and Infectious Diseases (NIAID) Fellowship 1-R38-AI174255-01 (EEH).
- American Heart Association postdoctoral fellowship 23DIVSUP1074485 (RL).
- NIH postdoctoral fellowship T32-HL160520 (EAH).
- German Research Foundation grant 209933838, SFB 1052 (project B1) (MB).
- Deutsches Zentrum für Diabetesforschung grant 82DZD00601 (MB).

## Supplementary Material

Supplemental data

Unedited blot and gel images

Supporting data values

## Version 1 10/28/2025

In-Press Preview

## Version 2 01/02/2026

Electronic publication
