# Supplementary material for: Secretory kinase FAM20C triggers adipocyte dysfunction, inciting insulin resistance and inflammation in obesity
Source: J Clin Invest. 2025 Oct 28;136(1):e191075. doi: 10.1172/JCI191075 (PMC12721910; doi:10.1172/JCI191075)
Supplement: Supplemental data [file jci-136-191075-s258.pdf]

Supplementary Figure S1: Related to Figure 1

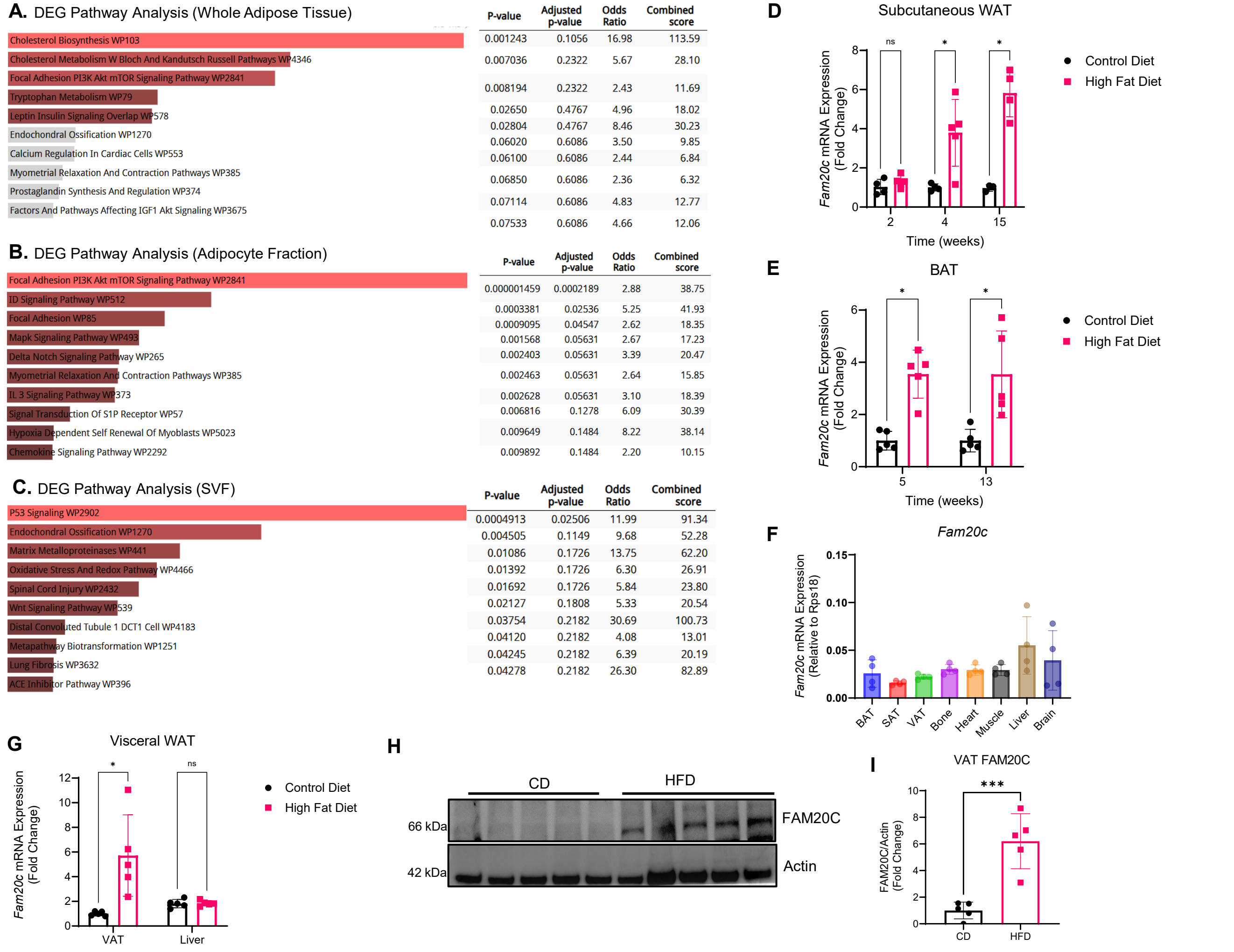

A. DEG Pathway Analysis (Whole Adipose Tissue)

Pathway	P-value	Adjusted p-value	Odds Ratio	Combined score
Cholesterol Biosynthesis WP103	0.001243	0.1056	16.98	113.59
Cholesterol Metabolism W Bloch And Kandutsch Russell Pathways WP4346	0.007036	0.2322	5.67	28.10
Focal Adhesion PI3K Akt mTOR Signaling Pathway WP2841	0.008194	0.2322	2.43	11.69
Tryptophan Metabolism WP79	0.02650	0.4767	4.96	18.02
Leptin Insulin Signaling Overlap WP578	0.02804	0.4767	8.46	30.23
Endochondral Ossification WP1270	0.06020	0.6086	3.50	9.85
Calcium Regulation In Cardiac Cells WP553	0.06100	0.6086	2.44	6.84
Myometrial Relaxation And Contraction Pathways WP385	0.06850	0.6086	2.36	6.32
Prostaglandin Synthesis And Regulation WP374	0.07114	0.6086	4.83	12.77
Factors And Pathways Affecting IGF1 Akt Signaling WP3675	0.07533	0.6086	4.66	12.06

B. DEG Pathway Analysis (Adipocyte Fraction)

Pathway	P-value	Adjusted p-value	Odds Ratio	Combined score
Focal Adhesion PI3K Akt mTOR Signaling Pathway WP2841	0.000001459	0.0002189	2.88	38.75
ID Signaling Pathway WP512	0.0003381	0.02536	5.25	41.93
Focal Adhesion WP85	0.0009095	0.04547	2.62	18.35
Mapk Signaling Pathway WP493	0.001568	0.05631	2.67	17.23
Delta Notch Signaling Pathway WP265	0.002403	0.05631	3.39	20.47
Myometrial Relaxation And Contraction Pathways WP385	0.002463	0.05631	2.64	15.85
IL 3 Signaling Pathway WP373	0.002628	0.05631	3.10	18.39
Signal Transduction Of S1P Receptor WP57	0.006816	0.1278	6.09	30.39
Hypoxia Dependent Self Renewal Of Myoblasts WP5023	0.009649	0.1484	8.22	38.14
Chemokine Signaling Pathway WP2292	0.009892	0.1484	2.20	10.15

C. DEG Pathway Analysis (SVF)

Pathway	P-value	Adjusted p-value	Odds Ratio	Combined score
P53 Signaling WP2902	0.0004913	0.02506	11.99	91.34
Endochondral Ossification WP1270	0.004505	0.1149	9.68	52.28
Matrix Metalloproteinases WP441	0.01086	0.1726	13.75	62.20
Oxidative Stress And Redox Pathway WP4466	0.01392	0.1726	6.30	26.91
Spinal Cord Injury WP2432	0.01692	0.1726	5.84	23.80
Wnt Signaling Pathway WP539	0.02127	0.1808	5.33	20.54
Distal Convoluted Tubule 1 DCT1 Cell WP4183	0.03754	0.2182	30.69	100.73
Metapathway Biotransformation WP1251	0.04120	0.2182	4.08	13.01
Lung Fibrosis WP3632	0.04245	0.2182	6.39	20.19
ACE Inhibitor Pathway WP396	0.04278	0.2182	26.30	82.89

Figure S1 (Related to Figure 1): (A) Pathway analysis of DEGs from whole adipose tissue, (B) adipocyte fraction, and (C) SVF of HFD vs. CD fed mice. (D) *Fam20c* mRNA expression from subcutaneous WAT of CD and HFD fed mice at respective time points (n=3-5 per group). (E) *Fam20c* mRNA expression from brown adipose tissue (BAT) of CD and HFD fed mice at respective time points (n=5 per group). (F) *Fam20c* mRNA expression from various tissues including BAT, subcutaneous WAT (SAT), visceral WAT (VAT), bone, heart, muscle, liver, and brain from WT mice on a CD (n=4 per group). (G) *Fam20c* mRNA expression from visceral WAT and liver of mice fed either a CD or HFD for 12 weeks (n=5 per group). (H) Representative western blot images and (I) quantification of FAM20C protein levels in VAT of mice fed either a CD or HFD for 12 weeks (n=5 per group). **p<0.05, ***p<0.001 by unpaired Student's t-test.*

Supplementary Figure S2: Related to Figure 1 and Figure 2

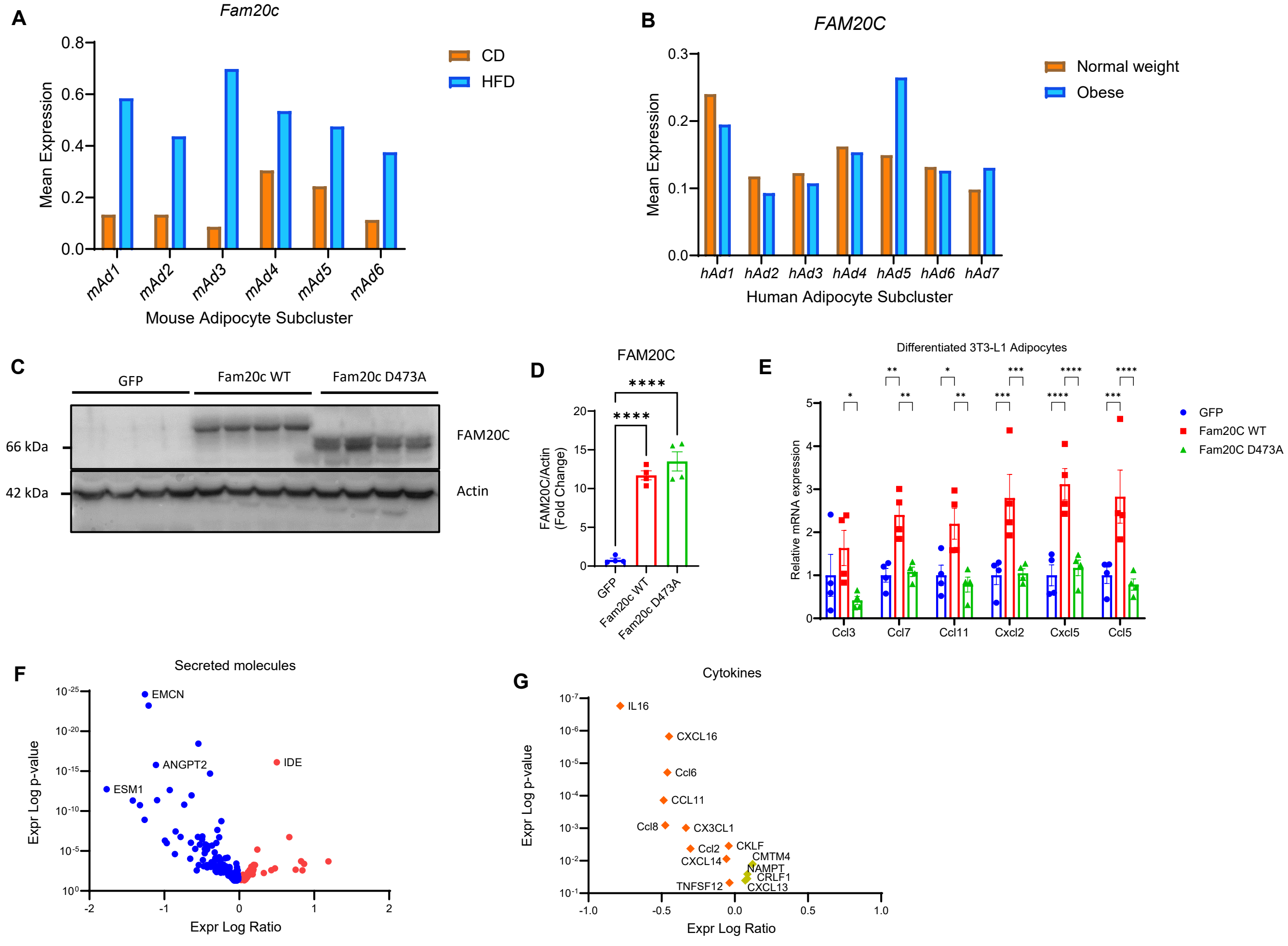

Figure S2 (Related to Figure 1 and 2): (**A**) Relative *Fam20c* expression in various mouse adipocyte subclusters from CD vs. HFD, and (**B**) Relative *FAM20C* expression in various human adipocyte subclusters from normal weight vs. obese individuals analyzed from publicly available single-nuclei RNA-seq dataset (PMID: 35296864). (**C**) Representative western blot images and (**D**) quantification of FAM20C protein levels in primary differentiated adipocytes transduced with respective viral constructs (n=4 per group). (**E**) Relative mRNA expression of proinflammatory genes in differentiated 3T3-L1 adipocytes transduced with respective viral constructs (n=4 per group). (**B**) Volcano plot of genes filtered for 'secreted molecules' that are differentially regulated by *Fam20c*. (**C**) Volcano plot of genes filtered for 'cytokines' that are differentially regulated by *Fam20c*. **p<0.05, **p<0.01, ***p<0.001, ****p<0.0001 by two-way ANOVA followed by Bonferroni multiple comparison's test for E. One-way ANOVA for D.*

Supplementary Figure S3: Related to Figure 3

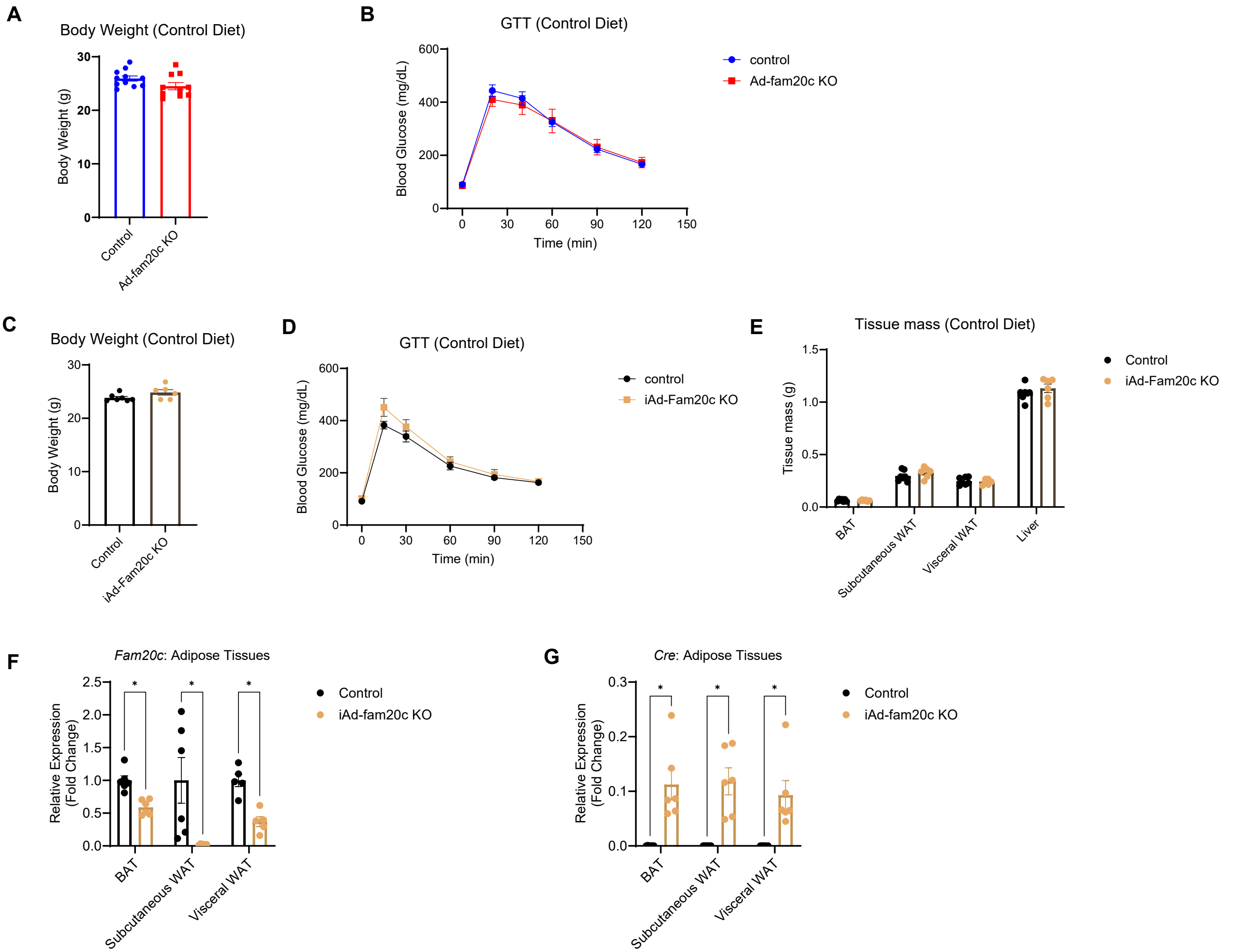

Figure S3 (Related to Figure 3): (**A**) Body weights and (**B**) Glucose tolerance test (GTT) for control and Ad-Fam20c KO mice fed a control diet (n=6-9 per group). (**C**) Body weights and (**D**) Glucose tolerance test for control and iAd-Fam20c KO mice fed a control diet (n=6-7 per group). (**E**) Weights of brown adipose tissue (BAT), subcutaneous white adipose tissue (WAT), visceral WAT, and liver from control and iAd-Fam20c KO mice fed a control diet (n=6-7 per group). (**F**) Relative mRNA expression for *Fam20c* and (**G**) *Cre* in various adipose depots including BAT, subcutaneous WAT, and visceral WAT of HFD-fed control and iAd-Fam20c KO mice following chronic *Fam20c* deletion (n=6 per group). **p<0.05 by unpaired Student's t-test.*

Supplementary Figure S4: Related to Figure 4

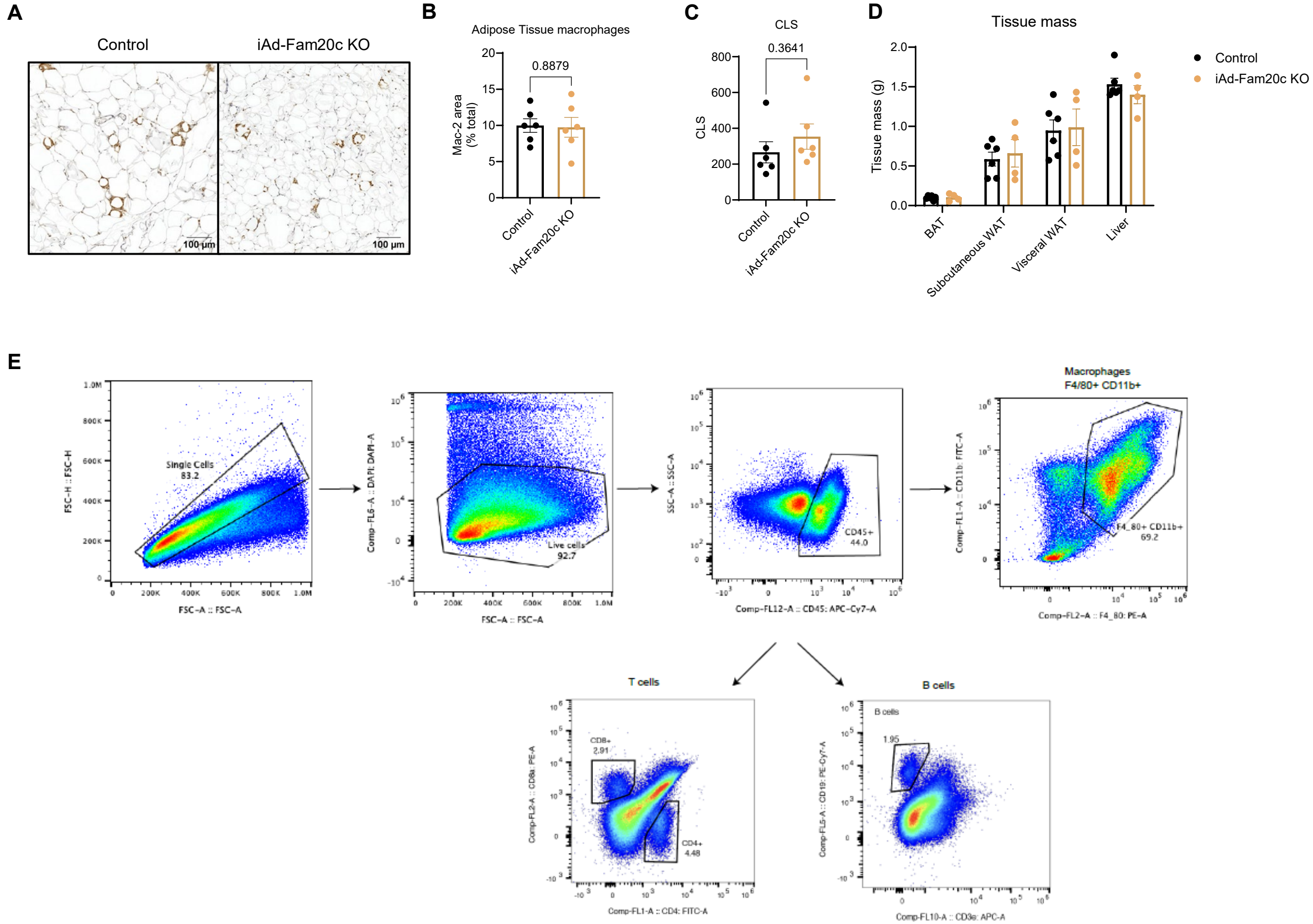

Figure S4 (Related to Figure 4): (**A**) Representative images of Mac-2-staining, (**B**) Quantification of Macrophage (Mac-2 stained) area represented as % of total adipose area, and (**C**) quantification of crown-like structures (CLS) represented as CLS per 10,000 adipocytes from visceral WAT sections of HFD-fed control and iAd-Fam20c KO mice following chronic *Fam20c* deletion (n=6 per group). (**D**) Mass of adipose tissue depots and liver from control and iAd-Fam20c KO mice fed HFD for 4 weeks followed by acute *Fam20c* deletion (n=4-6 per group). (**E**) Representative gating strategy for analyses of immune cell populations in the SVF of visceral WAT from control and iAd-Fam20c KO mice. F4/80 and CD11b were used to mark adipose tissue macrophages, CD19 was used to mark B cells, CD4 and CD8 were used to mark T-cells.

Supplementary Figure S5: Related to Figure 7

A Pathway Analysis of differentially expressed phosphopeptides

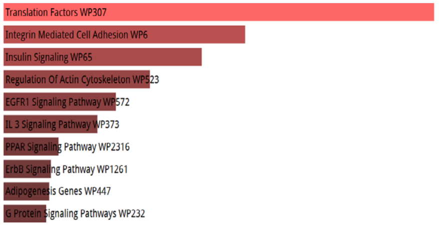

Term	P-value	Adjusted p-value	Odds Ratio	Combined score
Translation Factors WP307	5.009e-7	0.00003156	17.30	250.94
Integrin Mediated Cell Adhesion WP6	0.00006995	0.002203	7.61	72.77
Insulin Signaling WP65	0.0002174	0.004564	5.37	45.25
Regulation Of Actin Cytoskeleton WP523	0.0008418	0.01326	4.93	34.93
EGFR1 Signaling Pathway WP572	0.002048	0.02580	4.19	25.97
IL 3 Signaling Pathway WP373	0.003321	0.03487	5.32	30.36
PPAR Signaling Pathway WP2316	0.009177	0.07974	5.17	24.27
ErbB Signaling Pathway WP1261	0.01120	0.07974	6.93	31.11
Adipogenesis Genes WP447	0.01170	0.07974	3.87	17.21
G Protein Signaling Pathways WP232	0.01266	0.07974	4.69	20.47

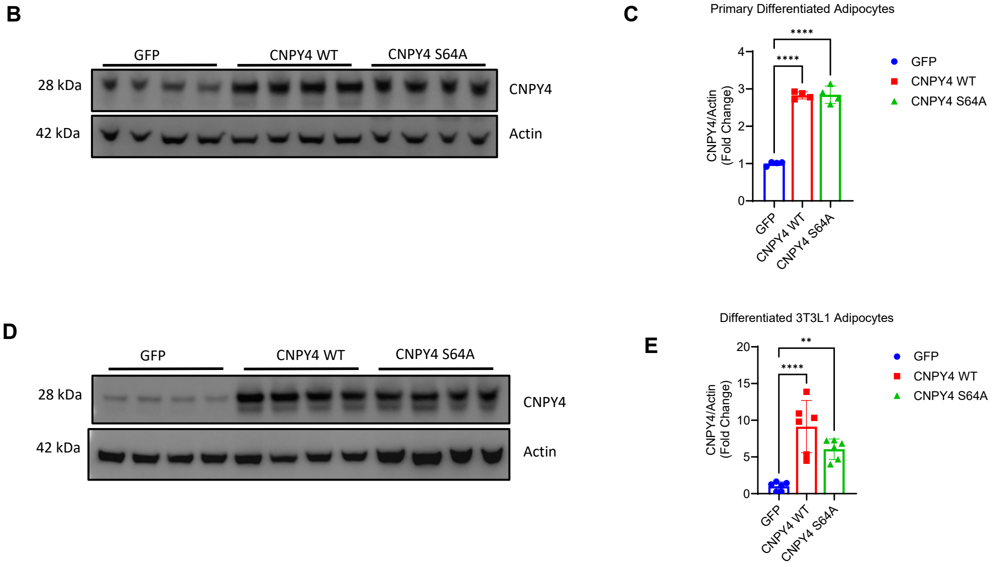

Figure S5 (Related to Figure 7): (**A**) Pathway Analysis of differentially expressed phosphopeptides from visceral WAT of HFD-fed control and Ad-Fam20c KO mice. (**B**) Representative western blot images and (**C**) quantification of FAM20C protein levels in primary differentiated adipocytes transduced with respective viral constructs (n=4 per group). (**D**) Representative western blot images and (**E**) quantification of FAM20C protein levels in differentiated 3T3L1 adipocytes transduced with respective viral constructs (n=4 per group). **p<0.05, **p<0.01, ***p<0.001, ****p<0.0001 by One-way ANOVA for C, E.*

Supplementary Figure S6: Related to Figure 8

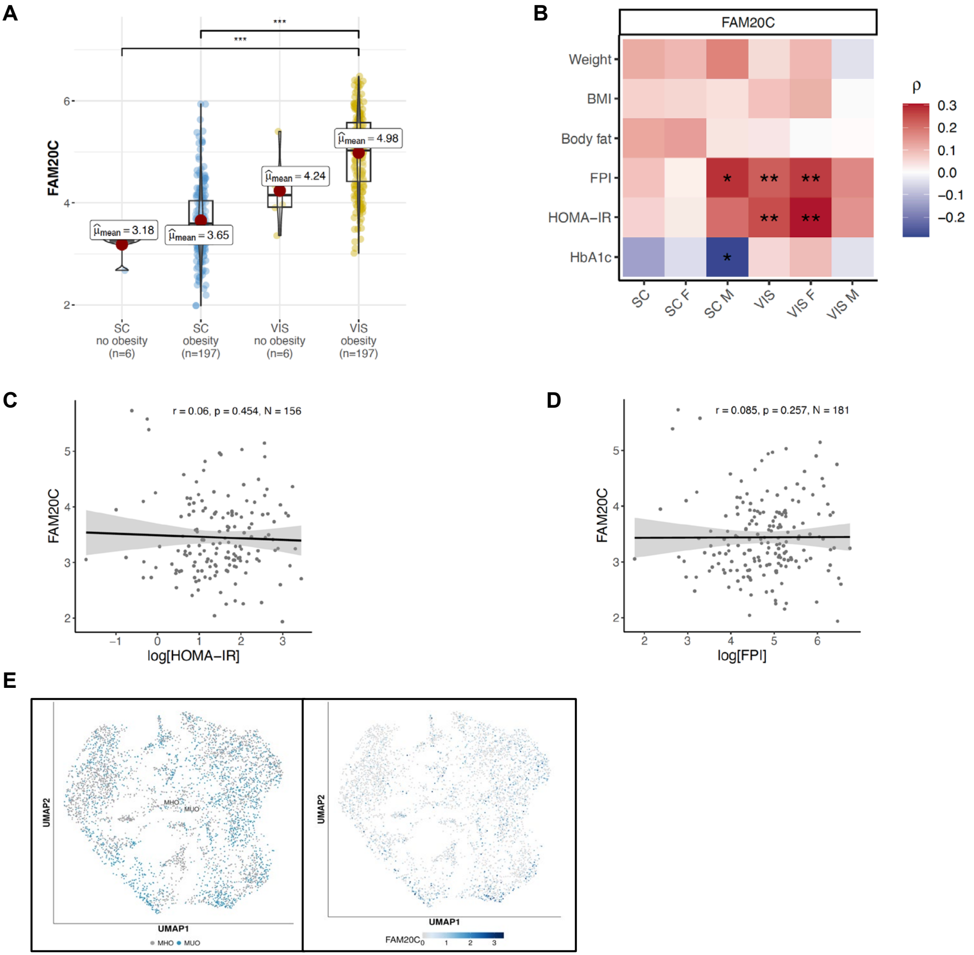

Figure S6 (Related to Figure 8): (**A**) *FAM20C* gene expression comparison for SC and VIS adipose tissues for patient subgroups not receiving antihyperglycemic medication with and without obesity (Welch's one way ANOVA; Games-Howell post-hoc Test). (**B**) *FAM20C* gene correlation analysis with metabolic parameters for patient subgroups not receiving antihyperglycemic medication (Spearman correlation coefficient with a confidence interval of 0.95). (**C**) Correlation of SC *FAM20C* gene expression in individuals not receiving antihyperglycemic medications with HOMA-IR. (**D**) Correlation of SC *FAM20C* gene expression in individuals not receiving antihyperglycemic medications with FPI. (**E**) UMAP plots showing visceral adipocyte *FAM20C* expression from single-nuclei RNA sequencing study in metabolically healthy obese (MHO) and metabolically unhealthy obese (MUO) individuals. *FPI: Fasting Plasma Insulin; HOMA-IR: Homeostasis Model Assessment of Insulin Resistance; C-D: Spearman correlation coefficient analysis with a confidence interval of 0.95.*

Supplement Table 1: qPCR Primer sequences.

Gene	Forward primer	Reverse primer
Fam20c	TGTGTCTCCAGCCAACAACAT	TCGCTTGTGGTAGGAACGAC
Il6	ACAACCACGGCCTTCCCTACTT	CACGATTTCCCAGAGAACATGTG
Ccl2	CCACTCACCTGCTGCTACTCAT	TGGTGATCCTCTTGTAGCTCTCC
Serpine1	TTCAGCCCTTGCTTGCCTC	ACACTTTTACTCCGAAGTCGGT
Vegfa	AGCACAGCAGATGTGAATGC	AATGCTTTCTCCGCTCTGAA
Ccl3	ACCATGACACTCTGCAACCA	CAGGAAAATGACACCTGGCT
Ccl5	CCCTCACCATCATCCTCACT	GAGCACTTGCTGCTGGTGTA
Ccl20	AGGCAGAAGCAGCAAGCAACTA	TGGATCAGCGCACACAGATTT
Il1b	TTTGACAGTGATGAGAATGACCT	GCTCTTGTTGATGTGCTGCTG
Tnf	CCCTCACACTCAGATCATCTTCT	GCTACGACGTGGGCTACAG
Ccl7	CCTGGGAAGCTGTTATCTTCA	AAGGCTTTGGAGTTGGGGTT
Ccl11	TGCTCACGGTCACTTCCTTC	CTTGAAGACTATGGCTTTCAGGGTG
Cxcl2	CCCAGACAGAAGTCATAGCCAC	TGGTTCTTCCGTTGAGGGAC
Cxcl5	TGCCCTACGGTGGAAGTCAT	AGCTTTCTTTTTGTCACTGCCC
Ccl5	TGCTCCAATCTTGCAGTCGT	GCAAGCAATGACAGGGAAGC
Cnpy4-1	TGAACGCAAGGGCTCACTAA	CGGTATTCGCACCTGCTTCT
Cnpy4-2	AGATACGCCAAGGGTCAAAGT	CATCGTCTCACACTGCTTCTTG
Cnpy4-3	CTGAGACGCGGCTAGAAGAG	CATCGTCTCACACTTGGCGT

Supplemental Methods:

Proteomic Sample Preparation for Differentiated Primary Adipocytes and Visceral White Adipose Tissue. Intracellular and secretome proteomics and phospho-proteomics sample preparation was conducted as previously described (61). For adipocytes, cells were washed 2x with Dulbecoo's PBS (Corning) and lifted using cell lifters (Corning) and centrifuged at 1100 r.p.m. for 4 minutes. The cell pellet was reconstituted in 1 mL RIPA lysis buffer (Thermo Pierce) containing protease and phosphatase inhibitors (Roche) and rotated end over end at 4 degrees Celsius for 20 minutes then centrifuged again to generate the soluble fraction of each cellular homogenate at 21,130 x g for 30 minutes at 4°C. Supernatants were transferred to clean 1.5 mL microcentifuge tubes, and soluble protein was quantified by micro BCA assay (Thermo Fisher Scientific - Pierce). Following quantification, protein equivalent to 200 µg of protein per sample was moved from each sample into a clean 1.5 mL tube. Following distribution of protein, each tube was brought to a final volume of 300 µL by addition of PBS with inhibitors, followed by precipitation with trichloroacetic acid (TCA) (Sigma) to a final concentration of 25%, vigorously vortexed and incubated on ice overnight. TCA precipitates were centrifuged at 21,130 x g for 30 minutes at 4°C, washed twice in 500 µL of ice-cold acetone, and centrifuged at 21,130 x g for 10 minutes after each wash. Following precipitation and washes, pellets were allowed to completely dry at room temperature. Dry pellets were re-suspended in 100 µL of 100 mM TEAB, 0.5% SDS and reduced with 9.5 mM tris-carboxyethyl phosphine (TCEP) for 60 minutes at 55°C. Following reduction of disulfide bonds with TCEP, the denatured protein mix was allowed to come to room temperature and centrifuged at 21,130 x g for 5 minutes at room temperature then alkylated with 4.5 mM

iodoacetamide (IAA) for 30 minutes in the dark at room temperature. After reduction and alkylation of disulfide bonds, the denatured protein mixture was precipitated out of solution by addition of 600 µL of ice-cold acetone (Millipore Sigma) and placed in the -20°C freezer overnight. The following day precipitated proteins were centrifuged at 8,000 x g for 10 minutes to pellet precipitated protein. Following centrifugation supernatant was decanted off and pellets were allowed to air-dry at room temperature. Once dry, protein pellets were reconstituted in 100 µL 100 mM TEAB and CaCl2 was supplemented to a final concentration of 1 mM, 1 µg of sequencing grade Trypsin (Promega) was added, and reactions were placed in the dark on a thermal mixer (Eppendorf) set to 37°C and shaking at 850 r.p.m. for 16 hours. The next day, digested samples were centrifuged at 21,130 x g for 10 minutes and proceeded to TMT labeling of digested samples. For visceral WAT tissue preparation all steps were congruent with above once soluble protein was generated

Proteomic Sample Preparation for Secretome of Differentiated Primary Adipocytes. For secretome proteomics and phospho-proteomics sample preparation, cells were washed 2x with Dulbecoo's PBS (Corning) and DMEM/F12 media without Fetal Bovine Serum was added and cultured for an additional 16 hours prior to media collection. Conditioned media was collected in a 50ml Conical tube and centrifuged at 2,000 RPM for 10 minutes to remove cellular debris. Following centrifugation, supernatant was decanted into a 50ml Luer Lok Syringe (Becton Dickinson) with an attached 0.45 µm PES filter and supernatant filtered into a clean 50ml conical. Soluble secreted protein was quantified by DC protein assay (Bio Rad). Following quantification, media equivalent to 200 µg of protein per sample was moved from each sample into clean 1.5 mL tubes.

Following distribution of protein, each tube was brought to a final volume of 300 µL by addition of PBS with inhibitors, followed by precipitation with trichloroacetic acid (TCA) (Sigma) to a final concentration of 25%, vigorously vortexed and incubated on ice overnight. TCA precipitates were centrifuged at 21,130 x g for 30 minutes at 4°C, washed twice in 500 µL of ice-cold acetone, and centrifuged at 21,130 x g for 10 minutes after each wash. Following precipitation and washes, pellets were allowed to completely dry at room temperature. Dry pellets were re-suspended in 100 µL of 100 mM TEAB, 0.5% SDS and reduced with 9.5 mM tris-carboxyethyl phosphine (TCEP) for 60 minutes at 55°C. Following reduction of disulfide bonds with TCEP, the denatured protein mix was allowed to come to room temperature and centrifuged at 21,130 x g for 5 minutes at room temperature then alkylated with 4.5 mM iodoacetamide (IAA) for 30 minutes in the dark at room temperature. After reduction and alkylation of disulfide bonds, the denatured protein mixture was precipitated out of solution by addition of 600 µL of ice-cold acetone (Millipore Sigma) and placed in the -20°C freezer overnight. The following day precipitated proteins were centrifuged at 8,000 x g for 10 minutes to pellet precipitated protein. Following centrifugation supernatant was decanted off and pellets were allowed to air-dry at room temperature. Once dry, protein pellets were reconstituted in 100 µL 100 mM TEAB and CaCl2 was supplemented to a final concentration of 1 mM, 1 µg of sequencing grade Trypsin (Promega) was added, and reactions were placed in the dark on a thermal mixer (Eppendorf) set to 37°C and shaking at 850 r.p.m. for 16 hours. The next day, digested samples were centrifuged at 21,130 x g for 10 minutes and proceeded to TMT labeling of digested samples.

Proteomic TMT Labeling, Fractionation, and Phosphopeptide Enrichment. For all proteomic experiments, TMT labeling was performed generally as per manufacturer's protocol (Thermo Scientific). Briefly, each TMT tag was re-suspended in 41 µL anhydrous acetonitrile with intermittent vortexing for 10 minutes. Following resuspension, 41 µL was added to corresponding protein digests and labeling reaction was allowed to proceed for 1 hour at room temperature. Reactions were quenched by addition of 8 µL of 5% hydroxylamine in 100 mM TEAB and incubated for 15 minutes. Labeled temperature fractions were pooled, desalted on 1cc/50 mg C18 SepPAK columns (Waters # WAT054955) on a vacuum manifold and desalted peptides were dried down in a speedvac. Dried, labeled peptides were re-dissolved in 200 µL of 40% acetonitrile, 6% TFA in water before phosphopeptide enrichment with Titansphere 5 µm TiO_2 beads (GL Sciences) and 10 µL (5% of the total volume) was removed for bulk proteomic analysis. For phospho-enrichments, Titansphere TiO_2 beads (GL Sciences) were reconstituted in buffer containing 80% acetonitrile, 6% TFA, and 2,5-dihydroxybenzoic acid (20 mg/mL) and rotated for 15 min at 25°C. Equal amount of beads slurry (~5:1 beads-to-peptide ratio based on concentration of peptides in 37°C aliquot) was added to reconstituted peptides and rotated for 20 mins 25°C. Beads were then washed twice with higher percentage of acetonitrile (10% and 40%) in 6% TFA and supernatant was removed by centrifugation at 500 x g for 2 min. Washed beads were then added to self-packed stage tip with C8 SPE (Sigma Aldrich) and washed once more with 60% acetonitrile in 6% TFA. Phosphopeptides were first eluted with 5% NH_4OH, then 10% NH_4OH, 25% acetonitrile, and dried in the speedvac. Dried phospho-peptides were reconstituted in 5% acetonitrile, 1% TFA, desalted with self-packed stage tip with C18 SPE (Sigma Aldrich), and dried with

speedvac once more in parallel with the 10 µL unenriched peptide aliquot removed prior to enrichment. Dried bulk and phospho-peptides were reconstituted in 300 µL of 0.1% TFA in H2O, high-pH reverse phase spin-columns (Thermo fisher scientific - Pierce) were equilibrated, and samples fractionated per manufacturer's instructions into 8 fractions, 2 washes and a flow-through fraction (11 total) for each layer of analysis. The final processed phospho-peptides were reconstituted in 5% acetonitrile, 0.1% TFA in water for LC-MS^3 analysis.

LC-MS^3 Analysis and Data Acquisition. High-pH reverse-phase fractions were run on a 4-hour instrument method with an effective linear gradient of 180 minutes from 5% to 25% mobile phase B with the following mobile phases: A: 0.1% formic acid in H2O, B: 80% acetonitrile/0.1% formic acid in water on a 50 cm Acclaim PepMap RSLC C18 column (Thermo Fisher Scientific #164942) operated by a Dionex ultimate 3000 RSLC nano pump with column heating at 50˚C connected to an Orbitrap Fusion Lumos. Briefly, the instrument method was a data-dependent analysis and cycle time set to 3 seconds, total. Each cycle consisted of one full-scan mass spectrum (400-1500 m/z) at a resolution of 120,000, RF Lens: 60%, maximum injection time of 100 ms followed by data-dependent MS/MS spectra with precursor selection determined by the following parameters: AGC Target of 4.0e5, maximum injection time of 100 ms, monoisotopic peak determination: peptide, charge state inclusion: 2-7, dynamic exclusion 10 sec with an intensity threshold filter: 5.0e3. Data-dependent MS/MS spectra were generated by isolating in the quadrupole with an isolation window of 0.4 m/z with CID activation and corresponding collision energy of 35%, CID activation time of 10 ms, activation Q of 0.25, detector type Ion Trap in Turbo mode, AGC target of 1.0e4 and maximum injection time of 120 ms.

Data-dependent multi-notch MS^3 was done in synchronous precursor selection mode (SPS, multi-notch MS^3) with the following settings: Precursor selection Range; Mass Range 400-1200, Precursor Ion Exclusion Properties m/z Low: 18 High: 5, Isobaric Tag Loss Exclusion Properties: TMT. Number of SPS precursors was set to 10 and data-dependent MS^3 was detected in the Orbitrap (60,000 resolution, scan range 120-500) with an isolation window of 2 m/z HCD activation type with collision energy of 55%, AGC target of 1.2e5 and a maximum injection time of 150 ms. Raw files were parsed into MS^1, MS^2 and MS^3 spectra using RawConverter.

Data Analysis for phosphoproteomics. Data generated were searched using the ProLuCID algorithm in the Integrated Proteomics Pipeline (IP2) software platform. Mouse proteome data were searched using a concatenated target/decoy UniProt database (May 25, 2018, release). Basic searches were performed with the following search parameters: HCD fragmentation method; monoisotopic precursor ions; high resolution mode (3 isotopic peaks); precursor mass range 600-6,000 and initial fragment tolerance at 600 p.p.m.; enzyme cleavage specificity at C-terminal lysine and arginine residues with 3 missed cleavage sites permitted; static modification of +57.02146 on cysteine (carboxyamidomethylation), +229.1629 on N-terminal and lysine for TMT-10-plex tag; 4 total differential modification sites per peptide, including oxidized methionine (+15.9949), and phosphorylation (+79.9663) on serine, threonine, and tyrosine (only for phospho-enriched samples); primary scoring type by XCorr and secondary by Zscore; minimum peptide length of six residues with a candidate peptide threshold of 500. A minimum of one peptide per protein and half-tryptic peptide specificity were required. Non-unique peptides were included in search. Starting statistics were performed with a Δmass cutoff

= 10 p.p.m. with modstat, and trypstat settings. False-discovery rates of peptide (sfp) were set to 1%. TMT quantification was performed using the isobaric labeling 10-plex labeling algorithm, with a mass tolerance of 5.0 p.p.m. or less. Reporter ions 126.127726, 127.124761, 127.131081, 128.128116, 128.134436, 129.131417, 129.13779, 130.134825, 130.141145, and 131.13838 were used for relative quantification.
