# Supplementary material for: Secretory kinase FAM20C triggers adipocyte dysfunction, inciting insulin resistance and inflammation in obesity
Source: J Clin Invest. 2025 Oct 28;136(1):e191075. doi: 10.1172/JCI191075 (PMC12721910; doi:10.1172/JCI191075)
Supplement: Unedited blot and gel images [file jci-136-191075-s259.pdf]

Full unedited blot for Figure 2E

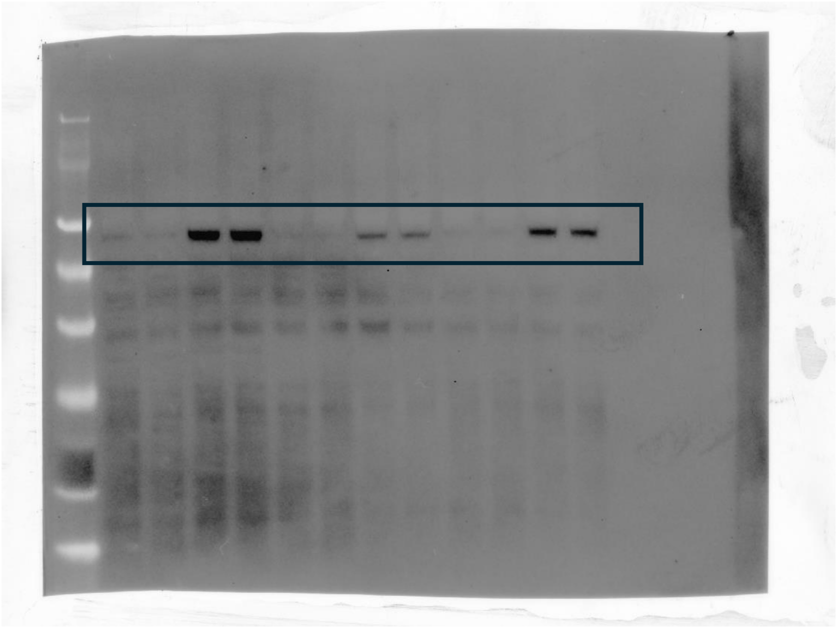

IB: P-Akt Ser473

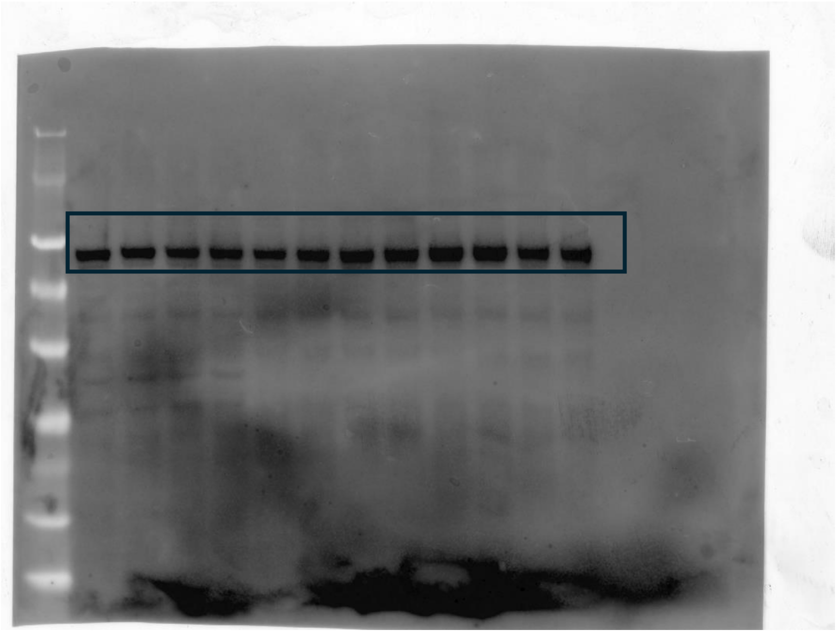

IB: Akt

Full unedited blot for Figure 4F

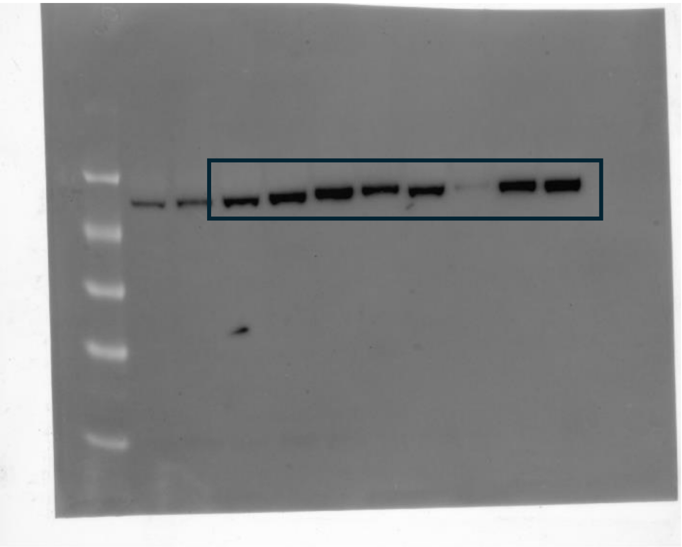

IB: P-Akt Ser473

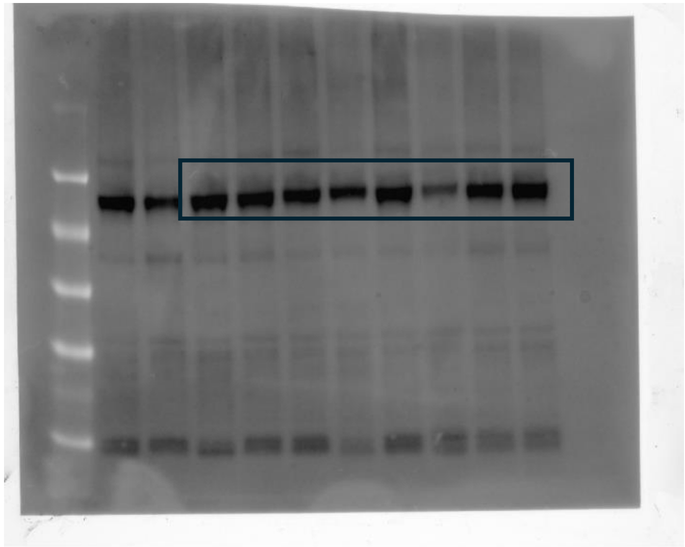

IB: Akt

Full unedited blot for Figure 4F

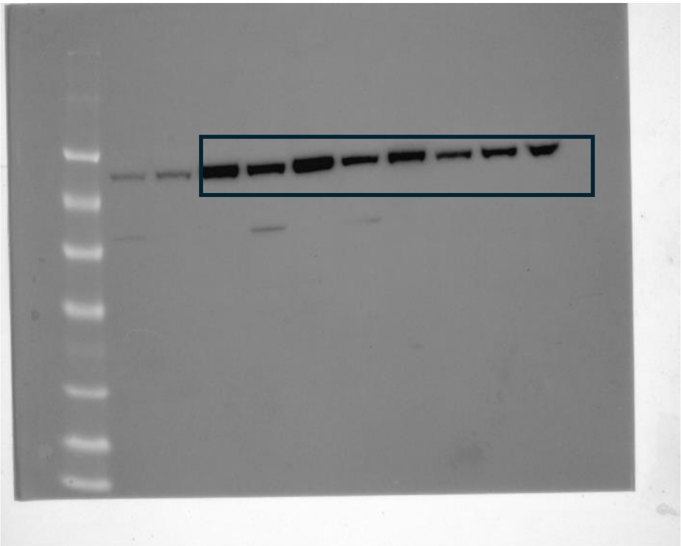

IB: P-Akt Ser473

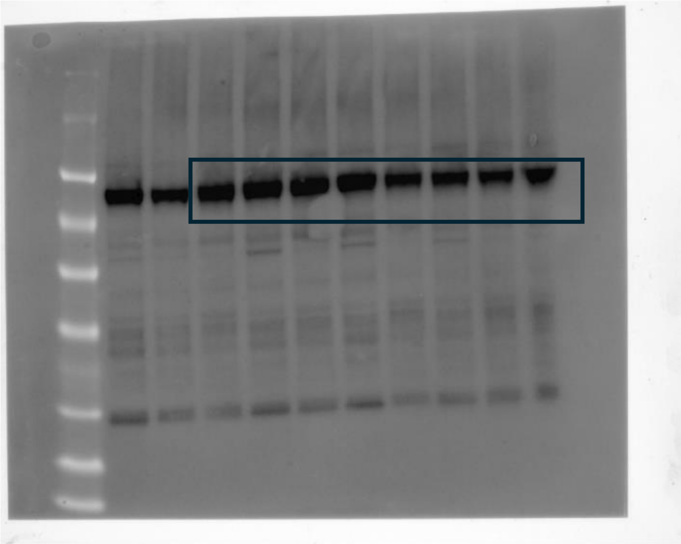

IB: Akt

Full unedited blot for Figure 4F

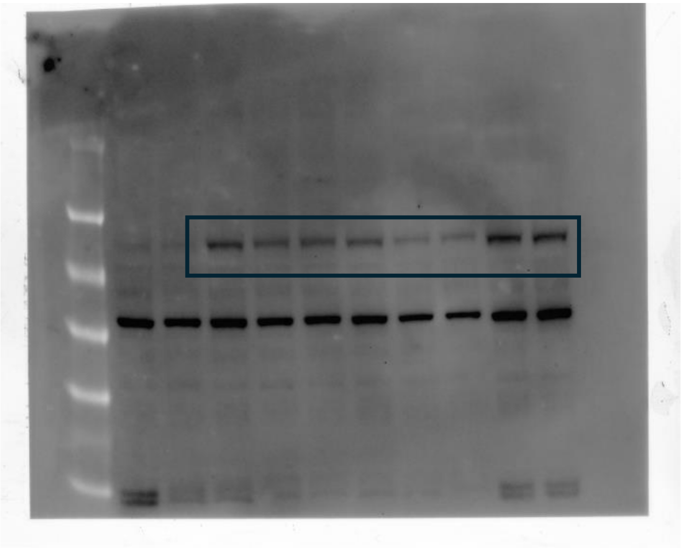

IB: P-Akt Ser473

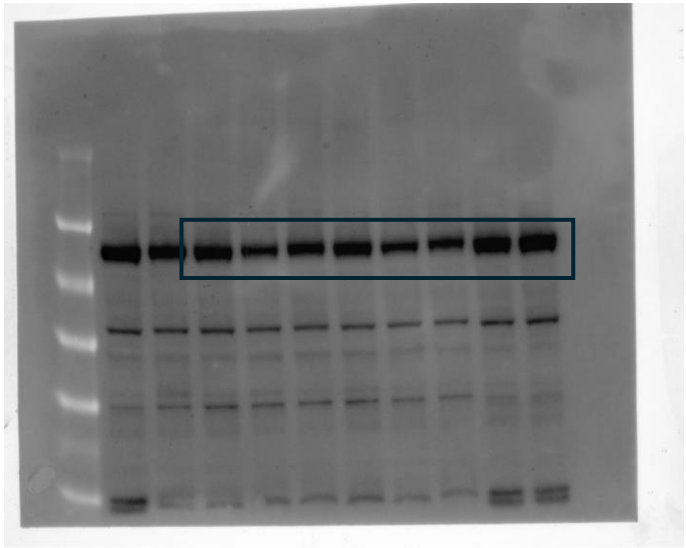

IB: Akt

Full unedited blot for Figure 4F

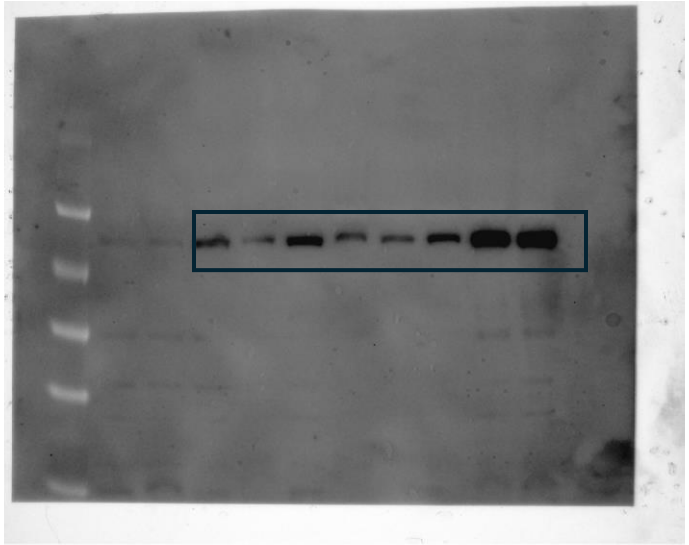

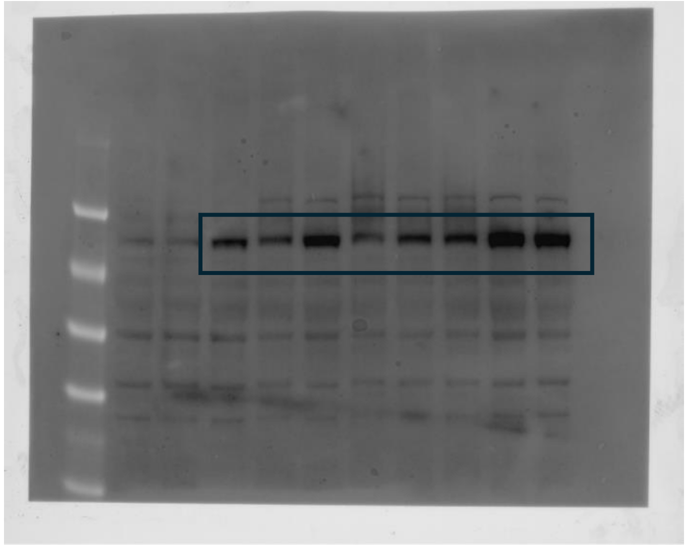

IB: P-Akt Ser473

IB: Akt

Full unedited blot for Figure 7F

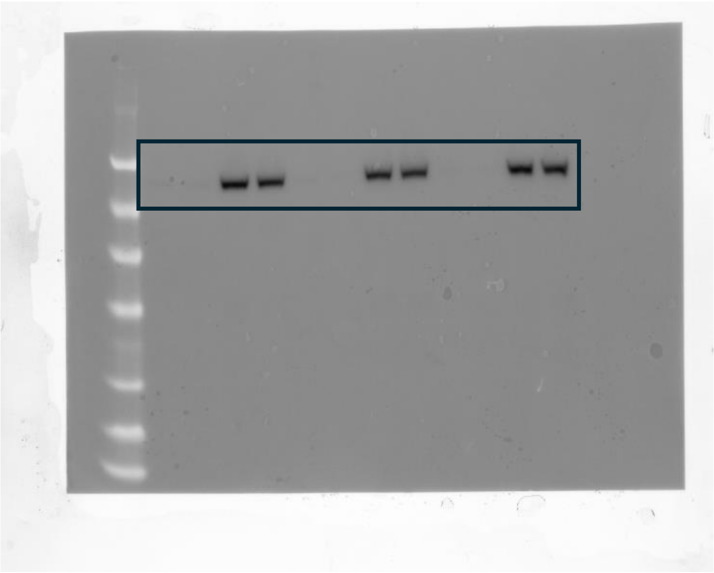

IB: P-Akt Ser473

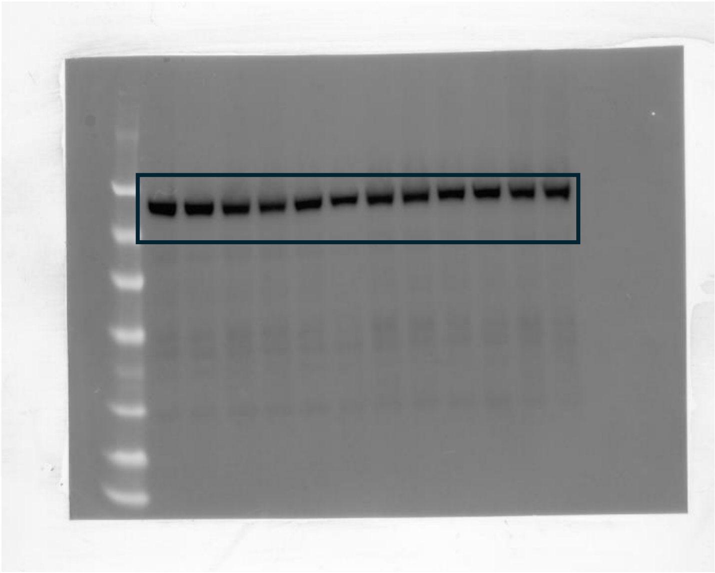

IB: Akt

Full unedited blot for Figure S1H

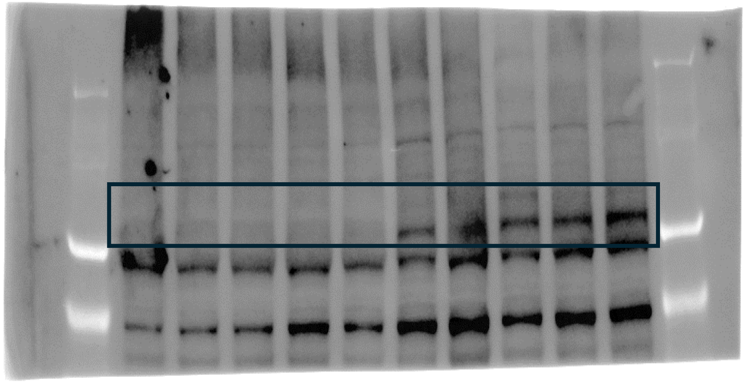

IB: FAM20C

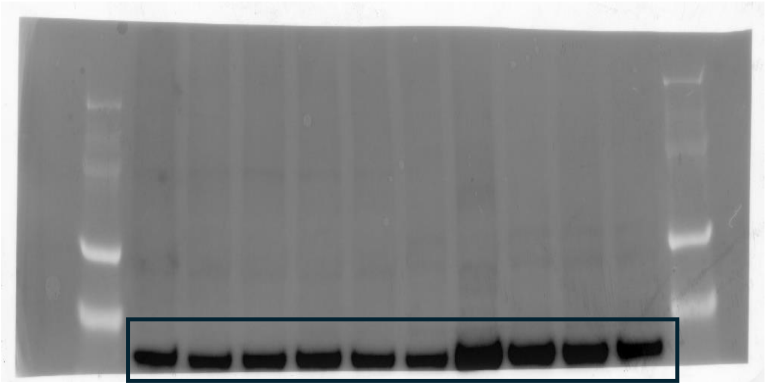

IB: Beta-Actin

Full unedited blot for Figure S2C

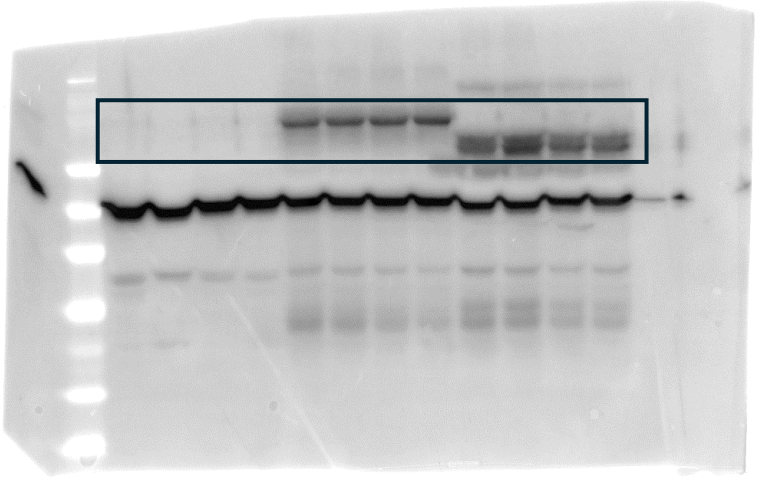

IB: FAM20C

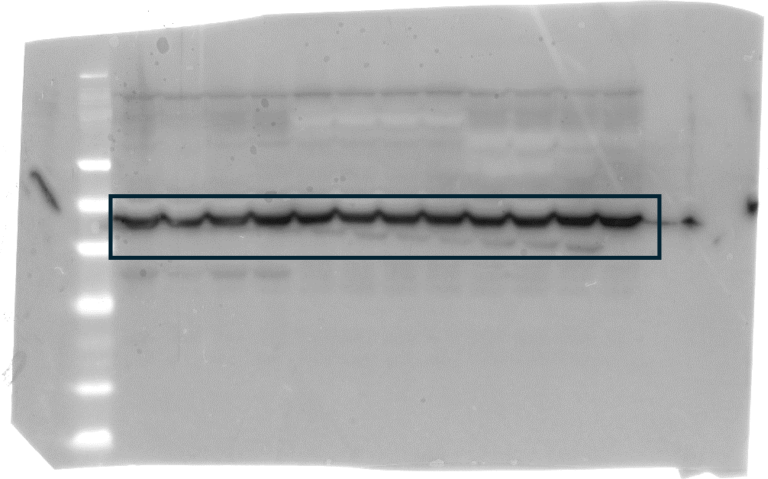

IB: Beta-Actin

Full unedited blot for Figure S7B

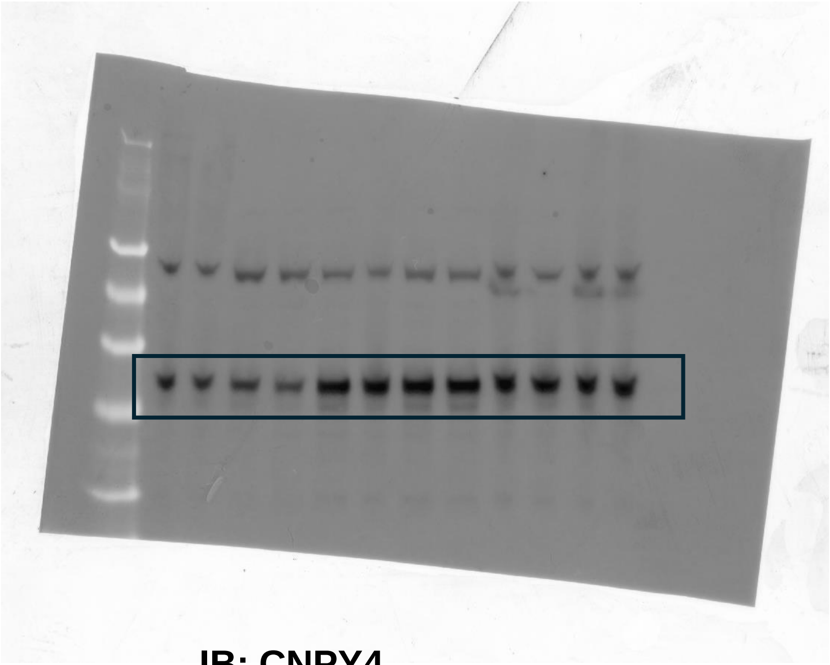

IB: CNPY4

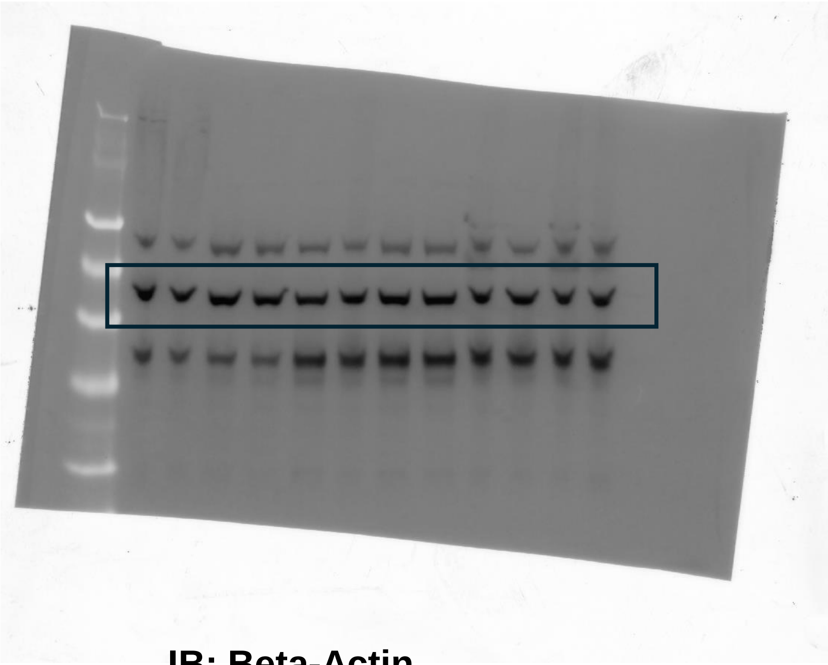

IB: Beta-Actin

Full unedited blot for Figure S7D

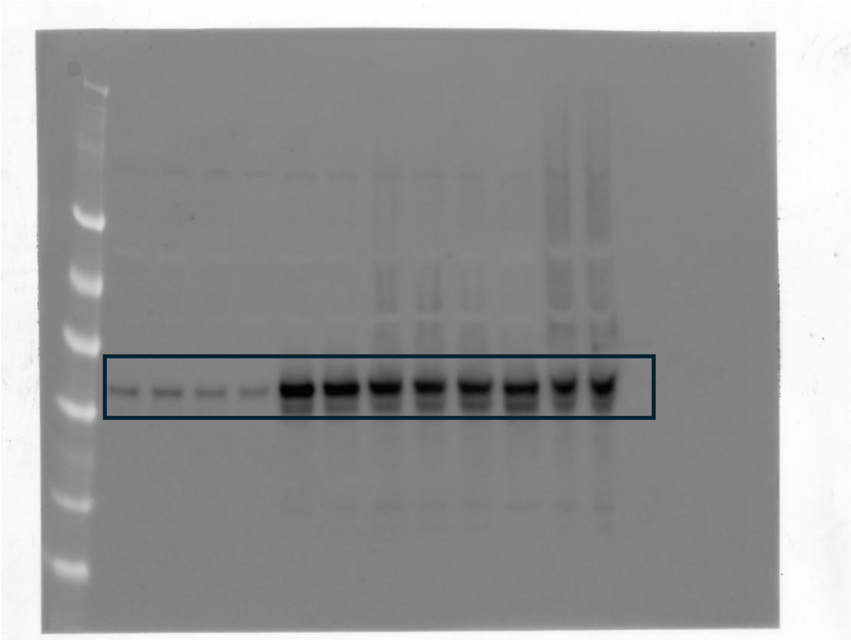

IB: CNPY4

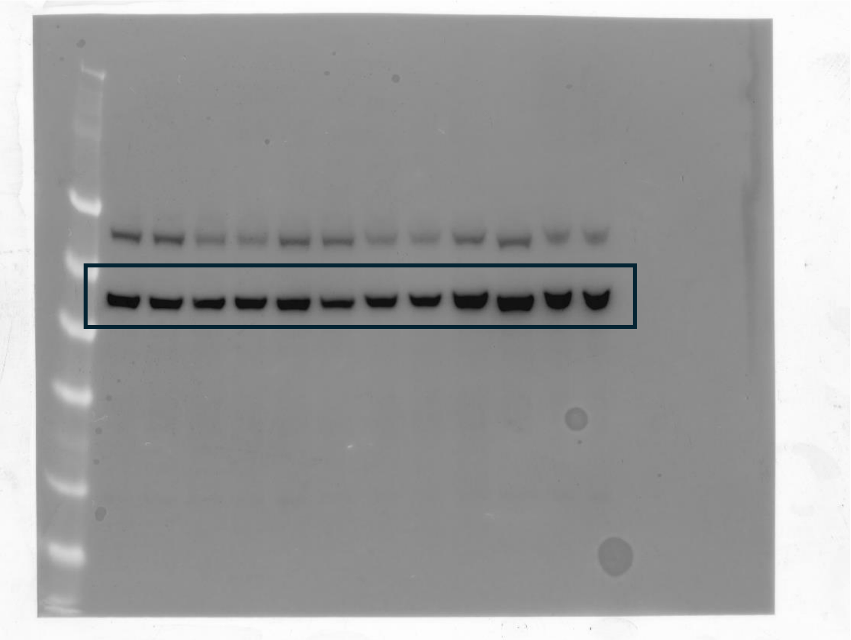

IB: Beta-Actin
